# The impact of hyperpolarization-activated cyclic nucleotide-gated (HCN) and voltage-gated potassium KCNQ/Kv7 channels on primary microglia function

**DOI:** 10.1186/s12974-020-01779-4

**Published:** 2020-04-06

**Authors:** Sabine Ulrike Vay, Lea Jessica Flitsch, Monika Rabenstein, Helena Monière, Igor Jakovcevski, Pavle Andjus, Dunja Bijelic, Stefan Blaschke, Helene Luise Walter, Gereon Rudolf Fink, Michael Schroeter, Maria Adele Rueger

**Affiliations:** 1 Department of Neurology, Faculty of Medicine and University Hospital, University Hospital of Cologne, Kerpener Strasse 62, 50924 Cologne, Germany; 2 Institute for Molecular and Behavioural Neuroscience and Center for Molecular Medicine Cologne, University of Cologne, Cologne, Germany; 3 Center for Laser Microscopy−CLM, Faculty of Biology, University of Belgrade, Belgrade, Serbia; 4 Cognitive Neuroscience, Institute of Neuroscience and Medicine (INM-3), Research Centre Juelich, Juelich, Germany

**Keywords:** Neuroinflammation, Cerebral ischemia, Ion channel, I_h_-current, ZD7288, XE-991, Microglia activation, Voltage sensor probes, Phagocytosis, Migration, Microglia phenotype, siHCN2

## Abstract

**Background:**

Microglia are essential to maintain cell homeostasis in the healthy brain and are activated after brain injury. Upon activation, microglia polarize towards different phenotypes. The course of microglia activation is complex and depends on signals in the surrounding milieu. Recently, it has been suggested that microglia respond to ion currents, as a way of regulating their activity and function.

**Methods and results:**

Under the hypothesis that HCN and KCNQ/Kv7 channels impact on microglia, we studied primary rat microglia in the presence or absence of specific pharmacological blockade or RNA silencing. Primary microglia expressed the subunits HCN1-4, Kv7.2, Kv7.3, and Kv7.5. The expression of HCN2, as well as Kv7.2 and Kv7.3, varied among different microglia phenotypes. The pharmacological blockade of HCN channels by ZD7288 resulted in cell depolarization with slowly rising intracellular calcium levels, leading to enhanced survival and reduced proliferation rates of resting microglia. Furthermore, ZD7288 treatment, as well as knockdown of HCN2 RNA by small interfering RNA, resulted in an attenuation of later microglia activation—both towards the anti- and pro-inflammatory phenotype. However, HCN channel inhibition enhanced the phagocytic capacity of IL4-stimulated microglia. Blockade of Kv7/KCNQ channel by XE-991 exclusively inhibited the migratory capacity of resting microglia.

**Conclusion:**

These observations suggest that the HCN current contributes to various microglia functions and impacts on the course of microglia activation, while the Kv7/KCNQ channels affect microglia migration. Characterizing the role of HCN channels in microglial functioning may offer new therapeutic approaches for targeted modulation of neuroinflammation as a hallmark of various neurological disorders.

## Background

Cerebral ischemia leads not only to acute tissue damage but also induces sustained neuroinflammation, which may persist for months after stroke [1–3]. Neuroinflammation eliminates degenerating tissue, thereby supporting a neuroprotective and restorative milieu that serves axonal regeneration, remyelination, and stem-cell-mediated tissue repair [4–6]. However, some of these immune processes may potentially be dysregulated, resulting in detrimental effects that promote secondary degeneration and, thus, hinder regeneration [7–10]. Hence, potentially helpful immune responses need to be tightly regulated, locally and timely, in order to adequately exert their beneficial effects and to avoid secondary damage [8].

Within the central nervous system (CNS), microglia are the resident immune cells that mediate and regulate inflammatory processes. Under physiological conditions, microglia are involved in the maintenance of cell homeostasis, surveying the microenvironment, and communicating with the neuronal tissue via extremely motile processes and protrusions [11]. Furthermore, microglia play an essential role in neuronal growth and maturation, function, and synaptic plasticity in both the developing and mature, healthy brain [4, 12–14]. After brain injury, microglia are activated and, depending on their activation phenotype, exert either neurotoxic or neuroprotective effects [15–17]. Although microglia are considered “non-excitable” cells of the CNS, they—like all mammalian cells—require active maintenance of their membrane potential to exert their functions [18].

In the past two decades, several studies provided evidence that variations in ion channel expression patterns of microglia (e.g., Kv1.3, Kir2.1, KCNN3, SK3, KCNN4, KCa3.1 channels) affect essential cell functions including activation, migration, proliferation, phagocytosis, cytokine secretion, and release of reactive oxygen species [19–25]. In particular, the expression levels of various ion channels depend on the functional state of microglia [23, 25–27], and some ion channels have been proposed as putative therapeutic targets to regulate neuroinflammation in neurological disorders [25, 27–29].

Assuming that further ion channels, which are present on neurons, would also be expressed in microglia and critically affect their function, we here for the first time assessed hyperpolarization-activated cyclic nucleotide-gated (HCN) channels and KCNQ/Kv7 channels on primary microglia in culture.

HCN channels are non-selective cation channels that mediate the hyperpolarization-activated current (h-current, I_h_). They are formed by the subunits HCN1-4, which are assembled as homo- or heterotetramers, and play a crucial role in rhythmic cells, e.g., cardiac rhythmic cells and neurons [30]. At subthreshold membrane potentials, HCN channels mediate a depolarizing (“pacemaker”) current that drives the cell membrane towards the action potential threshold. Mutations in neuronal HCN channel genes are associated with idiopathic generalized epilepsy [31] and infantile epileptic encephalopathy [32]. Although HCN channels are widely expressed in the CNS [33], very little is known about their role in non-excitable, non-neuronal cells of the CNS. Lau et al. reported that HCN channels play a substantial role in the proliferative capacity of mouse embryonic stem cells [34]. Furthermore, studies revealed an upregulation of HCN channels in reactive astrocytes after focal cerebral ischemia and global hypoxia in mice and rats, respectively [35, 36]. Notomi et al. examined HCN channel expression on osteoclasts and described its regulative effects on osteoclast differentiation [37].

The KCNQ/Kv7 channel—also known as M channel [38]—is a voltage-gated potassium channel that stabilizes the membrane potential in the presence of depolarizing currents by generating a steady voltage-dependent outward current [38]. The KCNQ genes 1 to 5 encode the corresponding proteins Kv7.1 to Kv7.5. In neurons, Kv7.2 and Kv7.3 heterodimerize with one another [39], and mutations in either gene can result in benign familial neonatal seizures or epileptic encephalopathy [40, 41]. In some neurons, Kv7.5 subunits heterodimerize with Kv7.3 subunits [42]. Kv7.1 is expressed in cardiomyocytes, and Kv7.4 is predominantly detected in vestibular hair cells and in the cochlea [43]. Similar to the HCN channel, studies about the assessment of KCNQ/Kv7 channels in non-neuronal CNS cells remain scarce.

## Material and methods

Animal procedures for tissue harvesting obeyed the German Federal Laws for Animal Protection. The local animal care committee (AZ UniKöln_Anzeige §4.16.021) approved the study. Animal procedures for tissue harvesting used for calcium imaging experiments followed the European Communities Council Directive (2010/63/EU) and Serbian Laboratory Animal Science Association for the protection of animals used for experimental and the scientific purposes and were approved by the Ethics Committee of the Faculty of Biology, University of Belgrade (EK-BF-2016/05).

### Microglia isolation and culture

Pure neonatal microglia cultures were obtained from the cortices of neonatal Wistar rats (P1-P3), as previously described in detail [17, 44, 45]. In brief, rat cortices were incubated in trypsin/EDTA solution (0.05% trypsin, 0.02% EDTA) for 15 min at 37 °C. Addition of the culture medium (Dulbeccos essential medium (DMEM) with the addition of 10% fetal calf serum (FCS), 1% penicillin/streptomycin, and 2 mM L-glutamine) stopped the reaction. The cortices were dissociated by repeated up- and down-pipetting. The resulting cell suspension was centrifuged at 1200 rpm for 2 min. Cells were re-suspended in DMEM (10% FCS, 1% penicillin/streptomycin, 2 mM L-glutamine) and grown at 37 °C with 5% CO_2_ for 8 to 10 days, while the culture medium was changed after the third day. This prolonged cultivation approach promoted a selective growth of astrocytes and microglia. To harvest pure microglia from this initial co-culture, culture flasks were shaken for 1 h at 250 rpm on an orbital shaker (37 °C) to detach microglia. The medium containing detached microglia was collected and immediately centrifuged for 2 min at 1200 rpm. The supernatant was removed, and the obtained pure microglia pellet was re-suspended in fresh culture medium and seeded into subcultures. Flasks were refilled with culture medium, and microglia harvesting was repeated for maximally three times at intervals of 3 days. All experiments were performed with these purified microglia cultures.

Twenty-four hours after seeding microglia in a subculture (50,000 cells per well on a 24-well plate or, for voltage-sensor-probe-experiments, 600,000 cells per well on a black 96-well plate with translucent bottom), cells were used for further experiments. Microglia were left unstimulated (control) or were stimulated with 10 ng/ml lipopolysaccharide (LPS derived from *E. coli* 0111: B4, Sigma Aldrich, St. Louis, USA) to activate microglia towards a pro-inflammatory phenotype, or alternatively stimulated with 50 ng/ml recombinant rat interleukin-4 (IL4; Peprotech, Hamburg, Germany) to polarize microglia to an anti-inflammatory phenotype [17]. To block HCN channels, microglia were treated with 10 μM or 30 μM ZD7288 (Sigma Aldrich, St. Louis, USA). To block KCNQ/Kv7 channels, microglia were treated with XE-991 (Sigma Aldrich, St. Louis, USA). For some experiments, combined LPS or IL4 plus ZD7288 or XE-991 (10 or 30 μM) were used. For migration assays, cells were treated immediately after seeding. In voltage-sensor experiments, ZD7288 (10 and 30 μM) or potassium (10 mM) was added 10 min before fluorescence measurement. For RNA transfection experiments, microglia were transfected 6 h after seeding and optionally co-treated with LPS (10 ng/ml) or IL4 (50 ng/ml) 18 h later. Experiments were generally stopped 24 h after treatment.

### RNA-transfection

Transfection with small interfering RNA (siRNA; 15 pmol/well) encoding HCN2 (Thermo Fisher Scientific; Silencer®Select Cat# 4390771, Table 1) and control siRNA (Thermo Fisher Scientific; negative control #1 and #2 siRNA; Silencer®Select Cat# 4390843 and 4390846) were performed using Lipofectamin® RNAiMAX (1.5 μl/well; Thermo Fisher Scientific) and Opti-MEM® medium (Opti-MEM; Thermo Fisher Scientific). Experiments were conducted according to the manufacturer’s protocol. Eighteen to twenty-four hours after transfection, cells were optionally stimulated with LPS (10 ng/ml) or IL4 (50 ng/ml). Note that previous control experiments showed no differences in survival (Live-Dead Assay, LDH release) and activation marker expression (iNOS, CD206) of untreated microglia compared to microglia treated with Lipofectamine® RNAiMAX/Opti-MEM-dilution without RNA (data not shown).

**Table 1:** Primers and parameters of RT-qPCR

RNA	Sequences forward/reversed 5`-3`	Temperature (°C) step 1/2/3	Duration (s) step 1/2/3	Accession number
Iba1	CCAGCGTCTGAGGAGCTATG/ CGTCTTGAAGGCCTCCAGTT	95/60/72	15/15/45	NM_017196.3
P2RY12	TTGCACGGATTCCCTACACC/ GGGTGCTCTCCTTCACGTAG	95/60/72	15/15/45	NM_022800.1
KCNK13	GAGAACGAAGGGCTCTACCG/ GAAGCACCCGCTATCCAGTT	95/60/72	15/15/45	NM_022293.1
HCN1	CAGAGCACTTCGGATCGTGA/ GGAGCAGCATCATGCCAATG	95/59.8/72	15/15/45	NM_053375.1
HCN2 N° 1	AGATCATCCTGGACCCCGAA/ GGATCTTGGTGAAACGCACG	95/59.8/72	15/15/45	NM_053684.1
HCN2 N° 2	ACCGGCATTGTTATTGAGGAC/ CCTCGGAGTCGATTCCCTTC	95/59.8/72	15/15/45	NM_053684.1
HCN3	GTGCAGTGGTTCGCATCTTC/ GGGAAGTCCTGCAGCATAGG	95/59.8/72	15/15/45	NM_053685
HCN4	CGGTCACCATCATCTAGCCC/ TGCCATAAAGGATGGCCGTT	95/59.8/72	15/15/45	NM_021658
KCNQ1	GTGGTCCAACCTGCAACAAC/ CTTCATCAGGGCCTGTCTGG	95/59.0/72	15/15/45	NM_032073.1
KCNQ2	CGTGGTATTCGGTGTTGAGT/ CTCCGAAGTGCAGACGTAG	95/57.8/72	15/15/45	NM_133322.1
KCNQ3	TCATGTCCGTCAACCACGAG/ ACCGCTTTTCCCTCATCCAG	95/59.0/72	15/15/45	NM_031597
KCNQ4	AATCCGCATAAGCATCTCCCA/ GAAGCTCCAGCTTTTCTGCAC	95/59.0/72	15/15/45	AF249748.1
KCNQ5	AAGACGGGAGACAGTACGGA/ TTCCTGACTCTTGCGCGATT	95/58.0/72	15/15/45	NM_001134643
iNOS	GCTTGTCTCTGGGTCCTCTG/ CTCACTGGGACAGCACAGAA	95/59.0/72	15/15/45	NM_012611.3
CD206	AACAAGAATGGTGGGCAGTC/ CCTTTCAGTCCTTTGCAAGC	95/56.0/72	15/15/45	NM_001106123.2
Ki67	TCTTGGCACTCACAGTCCAG/ GCTGGAAGCAAGTGAAGTCC	95/58.0/72	15/15/45	NM_001271366.1
RPL13a	TCTCCGAAAGCGGATGAACA/ CAACACCTTGAGGCGTTCCA		15/15/45	NM_173340.2
Small interfering RNA HCN2	GCACCGGCAUUGUUAUUGAtt UCAAUAACAAUGCCGGUGCga			

### Voltage sensor probes

**Fig. 1:**
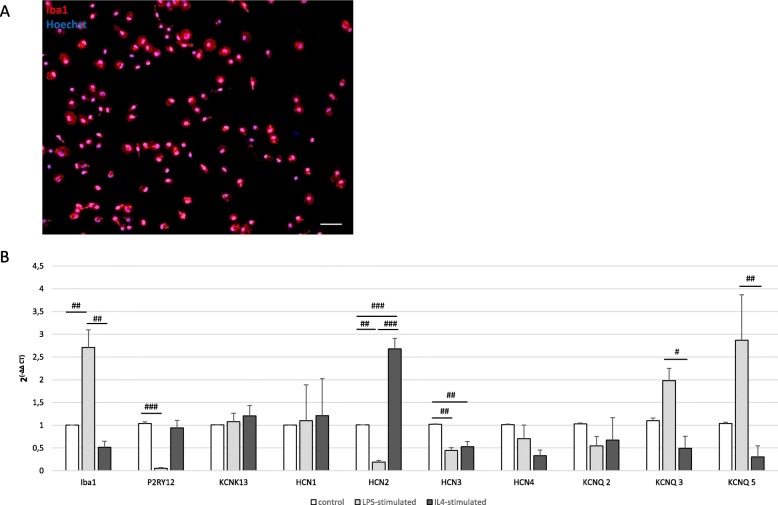
HCN channel and KCNQ/Kv7 channel expression on primary microglia. *#p* < 0.05, *##p* < 0.01, *###p* < 0.001 compared different experimental groups as marked by horizontal bar. **a** A representative image of primary microglia culture shows purity by staining of Iba1 (red). Hoechst stains all cell nuclei blue. Images were obtained with a fluorescence microscope, scale bars = 100 μm. **b** Expression of microglia marker Iba1 and P2RY12. Expression of microglial ion channels KCNK13, the HCN-subunits HCN1, HCN2, HCN3, and HCN4, as well as the KCNQ/Kv7 channel subunits KCNQ2, 3, and 5, on primary rat microglia in vitro measured by RT-qPCR. Microglia were untreated, activated with LPS (10 ng/ml) or IL4 (50 ng/ml; for Iba1: F(2, 6) = 23.571, *p* = 0.001, *ω* = 0.913; for P2YR12. F(2, 6) = 31.897, *p* = 0.001, *ω* = 0.934; for HCN2: F(2, 18) = 113.981, *p* < 0.0001, *ω* = 0.956; for HCN3: F(2, 8) = 22.573, *p* = 0.001, *ω* = 0.892; for KCNQ3: F(2, 11) = 11.674, *p* = 0.002, *ω* = 0.777; for KCNQ5: *H*(2) = 9.168, *p* = 0.01)

Experiments were conducted according to the manufacturer’s protocol (Thermo Fisher Scientific). After loading microglia with 30 μM CC2-DMPE and subsequently with 10 μM DiSBAC2(3)/per well in a buffer containing 4.5 mM KCl, fluorescence emission was detected with a plate reader (FLUOStar Omega, BMG Labtech, Ortenberg, Germany). For some experiments, ZD7288 (10 μM and 30 μM) or potassium (10 mM) were added 10 min before measurement. The optical filters were 405 +/− 10 nm for excitation and 460 +/− 10 nm and 570 nm +/− 10 nm for emission. Background signals were obtained using wells containing the cells and VSP-1 buffer only. The fluorescence emissions were recorded for 10 s to establish a baseline fluorescence ratio. To analyze the response on high potassium, a depolarizing solution containing 164.5 mM KCl was automatically added by the plate reader, and fluorescence emission were recorded for 30 s at 1 Hz. Raw data were blank-corrected, and emission rates were calculated as the mean value of the baseline emission at 460 +/− 10 nm divided by the mean value of baseline emission at 570 +/−10 nm (Fig. 1d). To assess the response rate upon high potassium, the emission rate after adding high potassium was divided by the baseline emission rate.

### Imaging of intracellular Ca^2+^

**Fig. 2:**
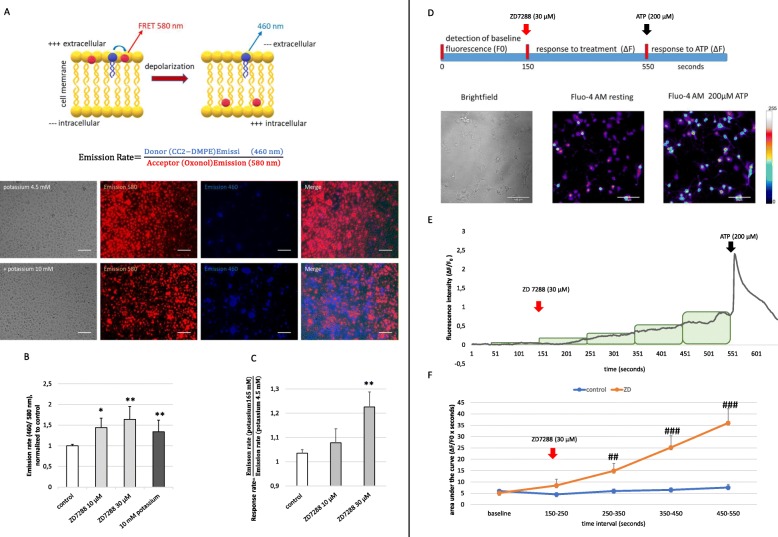
The impact of the HCN channel on the membrane potential and intracellular calcium concentration [Ca^2+^]_i._*p < 0.05, **p < 0.01, ***p < 0.001 compared to control; *#p* < 0.05, *##p* < 0.01, *###*p < 0.001 compared to the untreated experimental group measured at the same time interval. **a** HCN channel characterized by a fluorescence resonance energy transfer (FRET)-derived measurement of the membrane potential. The schematic illustration was modified from the description by Thermo Fisher Scientific. The FRET pair was composed of a highly fluorescent, mobile, voltage-sensitive acceptor oxonol (DiSBAC2) and a fluorescent, membrane-bound coumarin-phospholipid FRET donor (CC2-DMPE). In resting cells, both members of the FRET pair bind to the outer surface of the cell membrane, resulting in efficient FRET. When the cells are depolarized, the oxonol dye translocates to the inner surface of the cell membrane, resulting in diminished FRET. The emission rate (the ratio of the donor emission to acceptor emission) reports changes in the membrane potential and is low in polarized cells and increases in depolarized cells. Representative images of primary microglia supplied with VSPs. Fluorescence microscopy revealed the emission of 460 nm (blue) and 580 nm (red; scale bars = 100 μm). **b** Microglia exposed to the FRET pair CC2-DMPE and DISBAC2 were untreated or pre-treated with ZD7288 (10 and 30 μM) 10 min before the emission at 580 nm, and 460 nm was measured with a *BMG FLUOstar Omega* reader. Data reveal the calculation of the emission rate (460/580). Potassium served as a positive control. Data were normalized to the untreated control (*H*(3) = 17.188, *p* = 0.001). **c** Response rate was calculated as the fraction of the acute emission rate after treating cells with high potassium (165 mM KCl), and the emission rate before potassium was added. Data reveal the response rate of untreated microglia compared to microglia that were pretreated with 10 μM and 30 μM ZD7288 10 min before measurement started (*H*(2) = 9.379; *p* = 0.009). **d** To asses changes in the intracellular Ca^2+^ concentration, primary microglia were loaded with fluorescent Fluo-4 (Fluo-4 AM), and images were captured every second during the experiment. The schematic illustration gives an overview of the experimental setup. Thirty micromolar of ZD7288 and 200 μM ATP were added 150 s and 550 s after the start of the experiment, respectively. The time-resolved fluorescence intensity at baseline (F0), as well as the change of fluorescence (ΔF), was analyzed for each time point during measurement. Representative images depict primary microglia in the brightfield technique, as well as with fluorescence microscopy in untreated status and after treatment with 200 μM ATP. **e** Ca^2+^ response of one representative primary microglia cell was examined in the presence of 30 μM ZD7288 (after 150 s of baseline measurement) and subsequent ATP-application (200 μM, 400 s after ZD7288 application). Data are presented as ΔF/F0 for each time point. Rectangle depict 100-s intervals that were used to assess the **f** area under curve (AUC; ΔF/F0 × s). Data are shown as mean values of 38 controls and 38 ZD7288-treated cells. Only cells that responded to 200 μM ATP were used for data acquisition (MWU was performed for each time interval: 250–350 s, *p* = 0.004; 350–450 s as well as 450–550 s *p* < 0.0001)

Approximately 13,000 primary rat microglial cells were seeded on poly-l-lysine-coated (50 μg/ml) 7 mm coverslips, and were used for experiments 2 or 3 days later. Cells were incubated with calcium-sensitive probe Fluo-4 (5 μM, Molecular Probes) in a working buffer solution (140 mM NaCl, 5 mM KCl, 2 mM CaCl_2_, 2 mM MgCl_2_, 10 mM d-glucose, and 10 mM HEPES; pH 7.4; ~ 300 mOsM) for 30 min at room temperature. To allow de-esterification of the dye, cells were washed twice with the working buffer solution. Imaging was performed using an inverted fluorescent microscope (AxioObserver A1, Carl Zeiss, Germany) using water immersion objective LD LCI Plan-Apochromat 25x/0.8 (Carl Zeiss, Germany), “evolve”-EM 512 Digital Camera System (Photometrics, USA) and VisiFluor Calcium Ratio Imaging System. The excitation light source was a Xenon Short Arc lamp (Ushio, Japan) combined with a high-speed polychromator system (VisiChrome, Visitron Systems, Germany). The excitation light (480 nm) and the emission light passed through the Fluorescein isothiocyanate (FITC) filter set (Chroma Technology Inc., USA). Time-lapse images were obtained at 1-Hz sampling rate for up to 15 min per experiment with the VisiView high-performance imaging software (Visitron Systems, Germany). The cells were first perfused in a working buffer for 150 s for assessment of the baseline fluorescence, and then, the response of the cells to 30 μM ZD7288 was recorded for an additional 400 s, followed by the application of 200 μM ATP at the end of each experiment. Data analysis consisted of extracting average fluorescence intensities from regions of interest (ROIs) that were drawn around the cell soma according to the response to 200 μM ATP. For the background correction, additional ROIs were extracted from the background. Fluorescence data, representing the intracellular free calcium concentration were analyzed by custom-written the Matlab software (Math Works, Natick, MA, USA) and expressed as ΔF/F0, where ΔF represents the change in fluorescence emission and F0 the fluorescence baseline level. Additionally, the area under curve (AUC; ΔF/F0 × s) was calculated for five 100-s time intervals (see schematic rectangles Fig. 2e).

### Griess assay

NO-release from microglia was quantified by photometrical detection of NO using a Griess reagent kit (Biotium, Hayward, USA). Twenty-four hours after the last stimulation of microglia, the supernatant was collected, and the content of NO was measured following the manufacturer’s protocol. The optical density (OD) of each sample was measured at 548 nm in a plate reader (FLUOstar Omega, BMG LABTECH, Ortenberg, Germany). Mean values +/−standard error of the mean (SEM) were established among equally treated samples. Each experiment was conducted in triplicate.

### Enzyme-linked immunosorbent assays

Concentrations of insulin-like growth factor 1 (IGF1), a factor that is produced and secreted by microglia of the anti-inflammatory phenotype, were measured in the supernatant of microglia 24 h after the last stimulation using the mouse/rat IGF1 Quantikine enzyme-linked immunosorbent assays (ELISA) Kit (Cat# MG100, R&D systems, Minnesota Canada). Experiments were conducted according to the manufacturer’s protocol. The OD of each sample was measured using a plate reader. IGF1-concentrations of the samples were calculated based on standard curves. Mean values +/−SEM were established among equally treated samples. Each experiment was conducted in triplicate.

### Phagocytosis assay

Phagocytic activity of untreated and treated microglia was assessed using a phagocytosis assay (CytoSelect^TM^ 96-well Phagocytosis Assay, Cell Biolabs, San Diego, CA, USA) according to the manufacturer’s protocol. Briefly, phagocytic activity was measured by the amount of engulfed pre-labeled zymosan substrate uptake after 2 h of incubation by colorimetric detection after blocking external zymosan particles. The OD of each sample was measured at 405 nm using the plate reader. The resulting mean values +/−SEM were established among equally treated samples and compared to microglia not treated with zymosan. Each experiment was conducted in triplicate.

### Real-time quantitative PCR

RNA from cultivated cells was isolated 24 h after treatment using the GeneUP total RNA mini Kit (Biotechrabbit, Henningsdorf, Germany), following the manufacturer’s protocol. Total RNA concentration and purity were evaluated photometrically. Total RNA (10 ng) was converted to cDNA by reverse transcription with the QuantiTect reverse transcription Kit (Qiagen, Hilden, Germany) following the manufacturer’s recommendations. All primers used in this study were obtained from Biolegio (Nijmegen, The Netherlands). Primer sequences and PCR parameters are listed in Table 1. The samples were amplified and quantified on a Bio-Rad CFX Connect^TM^ real-time system (Hercules, CA, USA). PCR product integrity was evaluated by melting point analysis and agarose gel electrophoresis. The threshold cycle (CT) was normalized to ribosomal protein L13a (RPL13a; ΔCT). Data are depicted as 2^(−ΔΔCT)^. Real-time quantitative PCR (RT-qPCR) was performed in technical triplets, and each experiment was conducted in biological triplicate. Mean values +/− SEM were calculated for all samples.

### Live/dead assay

Cells were sown on 24-well plates (50,000 microglia cells/well). To assess the toxic effects of ZD7288, XE-991, and transfection with siRNA (with or without LPS and IL4, respectively), dead cells were stained with propidium iodide (Life Technologies, Darmstadt, Germany) 24 h after treatment. All cells, irrespective of viability, were counterstained with Hoechst 33342 (Life Technologies, Darmstadt, Germany). Representative pictures were taken using an inverted fluorescence phase-contrast microscope. Ten images per well were taken, and both Hoechst-stained, as well as propidium iodide-stained cells, were counted manually. The ratio of propidium iodide positives on total cell count provided the proportion of cell death. The experiment was performed in triplicate with four wells per condition. The resulting mean values +/− SEM were established among equally treated samples.

### Lactate dehydrogenase assay

In addition to the live/dead-assay, cell death was indirectly assessed by measuring lactate dehydrogenase (LDH) release to the media using a colorimetric assay (Pierce LDH assay kit, Thermo Scientific, Waltham, USA) 24 h after treatment with ZD7288, XE-991, and transfection with siRNA (plus/minus LPS and IL4, respectively). The experiment was performed according to the manufacturer’s protocol. The intensity of the red color formed in the assay was measured at a wavelength of 490 nm (FLUOstar Omega, BMG LABTECH, Ortenberg, Germany), being proportional to LDH activity and thus correlating with the number of damaged cells. Lysed cells served as a positive control. The experiment was performed in triplicate with four wells per condition. The resulting mean values +/− SEM were established among equally treated samples.

### Bromodeoxyuridine proliferation assay

Bromodeoxyuridine (BrdU) (Sigma Aldrich, St. Louis, USA) served to label and quantify proliferating microglia. Cells were sown on 24-well plates with glass coverslips inside the wells (50,000 microglia cells/well). Eighteen hours after treatment with ZD7288 and XE-991, 10 mM BrdU was added to each well. After 6 h of incubation, the experiment was stopped by cell fixation with 4% PFA, and cells were stained for incorporated BrdU (see below). The ratio of BrdU-positive cells on total cell count provided the proportion of proliferating cells. The experiment was performed in triplicate with four wells per condition. The resulting mean values +/− SEM were established among equally treated samples.

### Immunocytochemistry

The purity of microglia cultures was regularly verified, as described previously [17, 44]. The HCN subtypes were detected using the following antibodies: HCN1 (mouse monoclonal antibody, dilution 1:100, Cat# N70-28, UC Davis/NIH NeuroMab Facility), HCN2 (rabbit polyclonal antibody, dilution 1:1000, Cat# PA1-918), HCN3 (mouse monoclonal antibody, dilution 1:500, Cat# N75-175, UC Davis/NIH NeuroMab Facility), and HCN4 (mouse monoclonal antibody, dilution 1:500, Cat# N75-150, UC Davis/NIH NeuroMab Facility). Furthermore, KCNQ2/Kv7.2 (mouse monoclonal antibody, dilution 1:500, Cat# N75-079, UC Davis/NIH NeuroMab Facility) and KCNQ3/Kv7.3 (rabbit polyclonal antibody, dilution 1:100, Cat# PA1-930, Thermo Fisher Scientific, Waltham, USA) were used. Microglia were further stained against the ionized calcium-binding adapter molecule 1 (Iba1; goat polyclonal antibody, dilution 1:500, Cat# ab5076, Abcam, Milton, UK) and inducible nitric oxide synthase (iNOS, rabbit monoclonal antibody, dilution 1:500, Cat# 13120, Cell Signaling Technology, Boston, USA). To assess the proliferation rate via BrdU-incorporation, microglia were stained with anti-BrdU (mouse monoclonal, clone BU-33, dilution 1:200, Cat# B8434, Sigma Aldrich, St. Louis, USA). For visualization of primary antibodies, fluorescein-labeled anti-mouse immunoglobulin G (IgG) or anti-rabbit IgG were used (dilution 1:200, Cat# A11001, Cat# A11057, and Cat# A21206, Thermo Fisher Scientific, Waltham, USA). All cells were counterstained with Hoechst 33342.

Ten pictures of each sample were taken using an inverted fluorescence phase-contrast microscope, and at least 250 total cells per sample and experiment were counted manually. All immunocytochemical experiments were performed in triplicate. The resulting mean values +/− SEM were established among equally treated samples.

### Migration assay

Cell migration was analyzed via a trans-well migration assay using a modified Boyden chamber (CytoSelect^TM^ 24-Well Cell Migration Assay, 8 μM pore size, Cell Biolabs, Inc., San Diego, USA), following the manufacturer’s protocol. Briefly, freshly collected cells (50,000 microglia) were dissolved in a serum-free culture medium within the inserted upper chamber. As a chemo-attractive stimulus, a medium containing 10% FBS was added to the lower chamber. Either 10 μM or 30 μM of ZD7288 or XE-991, respectively, were added to the upper chamber of the modified Boyden chamber. Untreated cells served as a control. After 24 h, migrated cells on the lower side of the inserts were stained with crystal violet, extracted (extraction solution, Cell Biolabs, Inc., San Diego, USA), and quantified by photometrical detection (560 nm) on a plate reader (FLUOstar Omega, BMG LABTECH, Ortenberg, Germany). Mean values +/− SEM were established among equally treated samples. Each experiment was conducted in triplicate.

### Data processing and statistical analyses

Raw numerical data and graphics were processed with Microsoft Excel (Version 2016, Microsoft Corp., Seattle, WA, USA). Images were edited with Adobe Photoshop (Version CS2). Microsoft PowerPoint (Version 2016, Microsoft Corp.) and PDF creator (PDF24, pdfforge GmbH, Hamburg, Germany) were used to provide figures.

Statistical analyses were performed with IBM SPSS Statistics (Version 24, International Business Machines Corp. IBM, Armonk, USA). To determine whether variables met the assumptions of linear models, Kolmogorov-Smirnov or Shapiro-Wilk test for normal distribution and Levene’s test of variance homogeneity were performed. If all variables analyzed met the assumption of normality, one-way analysis of variance (ANOVA) was conducted to compare multiple groups. ANOVA was followed up by pairwise comparisons using Tukey-honest significant difference or Game’s Howell test, which was used for variables with unequal variances. In case parameters turned out to be not normally distributed, the non-parametric Kruskal-Wallis test was calculated and followed up by Bonferroni correction for multiple comparisons. Statistical significance was set at less than the 5% level (*p* < 0.05).

## Results

### Dynamic expression of HCN and KCNQ/Kv7 channels on primary microglia

The purity of our primary microglia culture was assessed by immunocytochemical staining for Iba1, which was expressed by > 99% of all cells (Fig. 1a). To obtain a pro-inflammatory phenotype, we treated microglia with LPS at 10 ng/ml [17]. To direct microglia towards an anti-inflammatory state, we stimulated microglia with IL4 at 50 ng/ml. We recently extensively characterized the effects of these substances on microglia activation [17]. Our first aim was to determine whether primary microglia expressed any HCN subunit (1–4) or Kv7 subunit (1–5) and whether the expression depended on the activation or polarization phenotype of microglia (Fig. 1b). To compare results, we first demonstrated the expression of characteristic microglia marker (Iba1 and P2YR12) that showed a significant regulation in dependency on the activation state of microglia. On the contrary, the expression of the well-known potassium channel THIK-1, which is encoded by KCNK13 and which exerts significant regulatory effects on the function of microglia [18], did not alter with microglial activation. RT-qPCR revealed the presence of all four HCN subunits in primary microglia, and interestingly, expression of HCN2 was significantly downregulated in LPS-treated microglia compared to untreated controls and, in turn, upregulated in microglia that was activated with IL4 (*p* < 0.01 and *p* < 0.001, respectively), whereas HCN3 expression was reduced in both activated microglia phenotypes (*p* < 0.01, Fig. 1b). Furthermore, microglia expressed KCNQ2, KCNQ3, and KCNQ5, encoding for Kv7.2, Kv7.3, and Kv7.5, respectively, at RNA expression level, while KCNQ1 and KCNQ4, encoding for Kv7.1 and Kv7.4, were not detectable by RT-qPCR. Interestingly, pro-inflammatory microglia expressed significantly higher levels of KCNQ3 and KCNQ5 compared to the anti-inflammatory phenotype of microglia (*p* < 0.05 and *p* < 0.01, respectively, Fig. 1b). Immunocytochemical staining confirmed stable protein expression of HCN1, HCN2, and HCN3 subunits, as well as Kv7.2 and Kv7.3, in primary microglia, whereas HCN4 subunit was almost not detectable (supplemental fig. 1).

### Functional characterization of HCN channels on primary microglia

To assess the influence of HCN channel blockade by ZD7288 (30 μM) on membrane potential in primary microglia, we used fluorescent voltage-sensor probes (VSPs), namely DiSBAC_4_(3) and CC2-DMPE. Figure 2 a schematically illustrates the fluorescence resonance energy transfer (FRET) mechanism of the used VSPs. The FRET donor (CC2-DMPE) is a membrane-bound, coumarin-phospholipid that binds to the exterior of the cell membrane. The FRET acceptor DiSBAC_4_(3) is a mobile, negatively charged, hydrophobic oxonol. At negative resting membrane potentials, the oxonol molecules bind to the extracellular membrane surface and, together with the FRET donor, provides a detectable energy transfer signal (emission at 580 nm). Upon cell depolarization, the oxonol dye translocates to the inner surface of the cell membrane, following the positive intracellular load, resulting in diminished FRET and increased emission of the FRET donor at 460 nm. Representative images show the enhanced emission of 460 nm fluorescence (blue) and the decrease of 580 nm fluorescence (red) emission after treating primary microglia with 10 mM potassium to induce depolarization (Fig.2a). The emission rate (the ratio of the donor emission to FRET emission) reports changes in membrane potential being low in polarized cells while increasing in depolarized cells (Fig. 2a, b). Blockade of HCN channels with ZD7288 (10 μM and 30 μM) 10 min before measuring the fluorescence signals led to a significant and dose-dependent increase of the emission rate on primary microglia compared to untreated controls (*p* < 0.05 and *p* < 0.01, respectively, Fig. 2b). Next, we assessed the acute change of the emission rate seconds after adding high potassium (165 mM KCl). We calculated the “response rate” as the fraction of the acute emission rate after treating cells with high potassium (165 mM KCl) and the emission rate before potassium was added (Fig. 2c). The response rate was not affected in microglia that had been pre-treated with 10 μM ZD7288 but significantly higher in microglia that had been pre-treated with ZD7288 in a dose of 30 μM (*p* < 0.01, Fig. 2c). These data suggest that treatment with 30 μM ZD7288 has (1) depolarizing effects on primary microglia and (2) strengthens the depolarizing effect of potassium.

Additionally, we assessed the effects of ZD7288 on changes in intracellular calcium ([Ca^2+^]_i_) that was monitored with the Ca^2+^-sensitive fluorescence dye Fluo-4. Application of 30 μM ZD7288 resulted in different Ca^2+^ responses (the fluorescence intensity after treatment (ΔF) was normalized to baseline fluorescence- ΔF/F0; supplemental fig. 2 A-E and representative graph in Fig. 2e). The majority of the cells did show either no reaction on ZD7288 (response below 75% of the response amplitude of untreated control cells, ~ 59% of cells; supplemental fig 1 A) or showed a slow but constant increase of [Ca^2+^]_i_ (~ 41%, supplemental fig. 2B and Fig. 2e). Only single cells showed [Ca^2+^]_i_ peaks with slow (supplemental fig. 2 C) or rapid (supplemental fig. 2D) reduction of [Ca^2+^]_i_, Ca^2+^ oscillations or Ca^2+^ peaks on top of an elevated Ca^2+^ level (supplemental fig. 2 E). Statistical analysis of the calculated area under the curve (AUC) in 100-s intervals after the ZD7288 application revealed a delayed but sustained increase of [Ca^2+^]_i_ compared to untreated control (*p* < 0.0001, Fig. 2f).

### Blockade of HCN and KCNQ/Kv7 channels affects essential functions of primary microglia without activating or polarizing effects

**Fig. 3:**
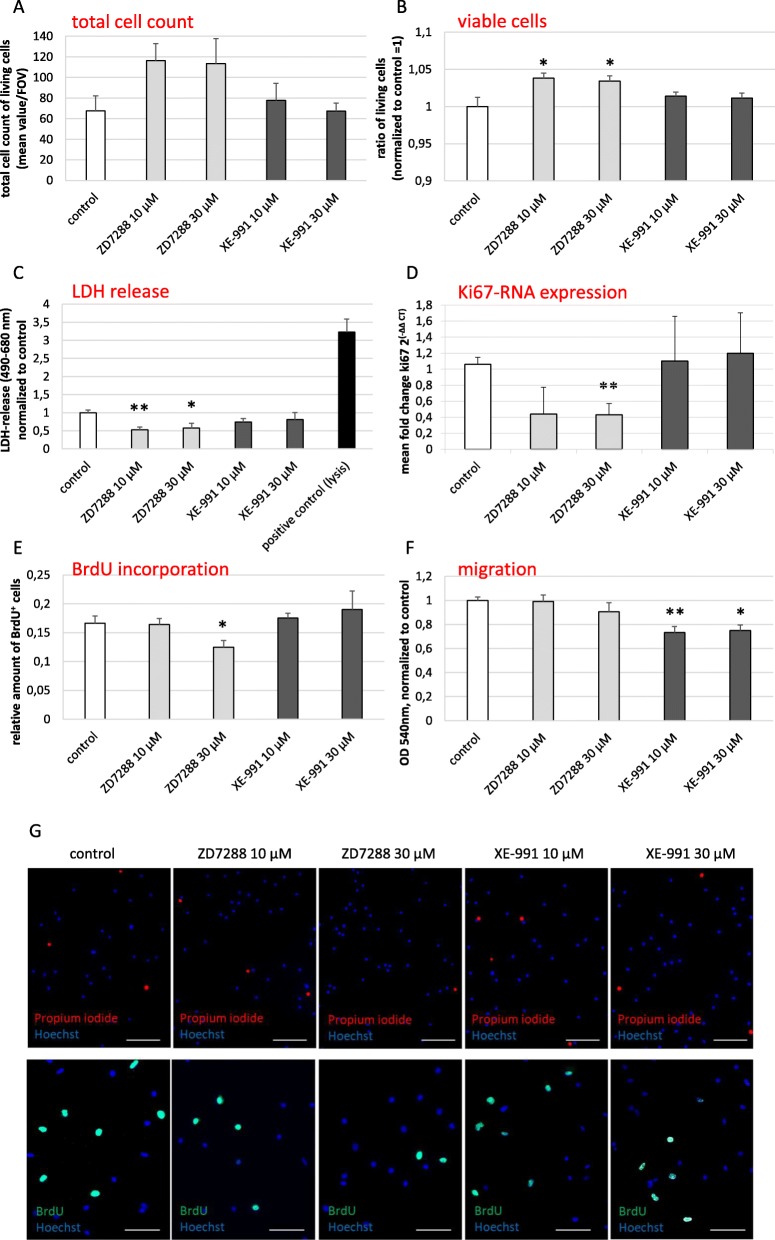
The impact of functional HCN and KCNQ/Kv7 channels on cell count, survival, and proliferation of primary microglia.*p < 0.05; **p < 0.01; ***p < 0.001 compared to controls. **a** The total number of viable cells was counted after blockade of the HCN channel with ZD7288 (10 μM and 30 μM, n.s.), and after blockade of KCNQ/Kv7 channel with XE-991 (10 μM and 30 μM). Data are shown as mean values per field of view (FOV). **b** Ratio of viable versus dead primary microglia subjected to ZD7288 and XE-991 as assessed by live/dead-assay (10 μM and 30 μM of each; F(4, 25) = 4.129, *p* = 0.011, *ω* = 0.375). Data are shown as a ratio of living cells normalized to control = 1 (absolute percentage of living cells of control = 93.5%). **c** Release of LDH was measured photometrically (LDH-assay) as a surrogate for cell death after treatment of microglia with ZD7288 or XE-991 (10 μM and 30 μM each). Lysed cells served as control (H(5) = 28.27, *p* < 0.0001; positive control is significant to all other conditions). **d** Proliferation rate was measured by Ki67 expression on RNA level by RT-qPCR after blockade of the HCN channel with ZD7288 and XE-991 (10 μM and 30 μM each; F(4,17) = 6.059, *p* = 0.003, *ω* = 0.48). **e** Proliferation rate after treatment with ZD7288 and XE-991 (10 μM and 30 μM each) was revealed by BrdU incorporation (F(4,19) = 3.319, *p* = 0.032, *ω* = 0.273). **f** Migration of microglia in the Boyden chamber assay under the influence of ZD7288 and XE-991(10 μM and 30 μM each; F(4, 47) = 4.846, *p* = 0.002, *ω* = 0.228). Data were blank-corrected and normalized to control. **g** Upper row: representative immunocytochemical images of the live/dead-assay. All cells regardless of viability stained by Hoechst (blue) and dead cells were identified by propidium iodide incorporation (red); scale bars = 100 μm. Lower row: representative images of the BrdU-proliferation assay are shown. Hoechst stains all cell nuclei blue, BrdU (green) identify proliferating cells. Scale bars = 50 μm

To explore the impact of pharmacological blockade of HCN and KCNQ/Kv7 channels on survival, proliferation potential, and migratory capacity of primary microglia, we cultivated cells for 24 h with either 10 μM or 30 μM ZD7288, or XE-991, respectively. The effects of the channel blockers on cell count and survival were assessed via live/dead-assay and LDH-release (Fig. 3a–c, g). ZD7288 in low (10 μM) and high dosage (30 μM) led to (1) an increase of the number of living cells in the cell culture by trend (n.s., Fig. 3a), (2) a significant increase in live/dead-cell ratio (plus 3.8% and 3.4%, compared to untreated control, *p* < 0.05 for 10 μM and 30 μM ZD7288, respectively, Fig. 3b, g), and (3) a reduced release of LDH compared to untreated control (*p* < 0.01 and *p* < 0.05, respectively, Fig. 3c). On the contrary, XE-991 exerted no effect on cell count or survival compared to untreated controls. The effect of microglia proliferation was assessed by RNA expression of Ki67, and by BrdU-incorporation. Treatment with 30 μM ZD7288 downregulated Ki67-expression to ~ 58% in comparison to untreated control (*p* < 0.05, Fig. 3d). Likewise, BrdU-incorporation was significantly reduced (12% proliferation rate in the ZD7288 (30 μM)-treated group compared to 17% in the untreated control group, *p* < 0.05, Fig. 3e, g). The blockade of KCNQ/Kv7 by XE-991 did not affect the microglia proliferation rate. As a crucial third function of microglia, their migratory potential was assessed by the Boyden chamber assay. Figure 3 f demonstrates that exposure to ZD7288 did not affect the migratory potential of microglia. Interestingly, XE-991 in low and high concentrations led to a significantly decreased migratory activity of microglia (*p* < 0.01 and *p* < 0.05, respectively, Fig. 3f).

**Fig. 4:**
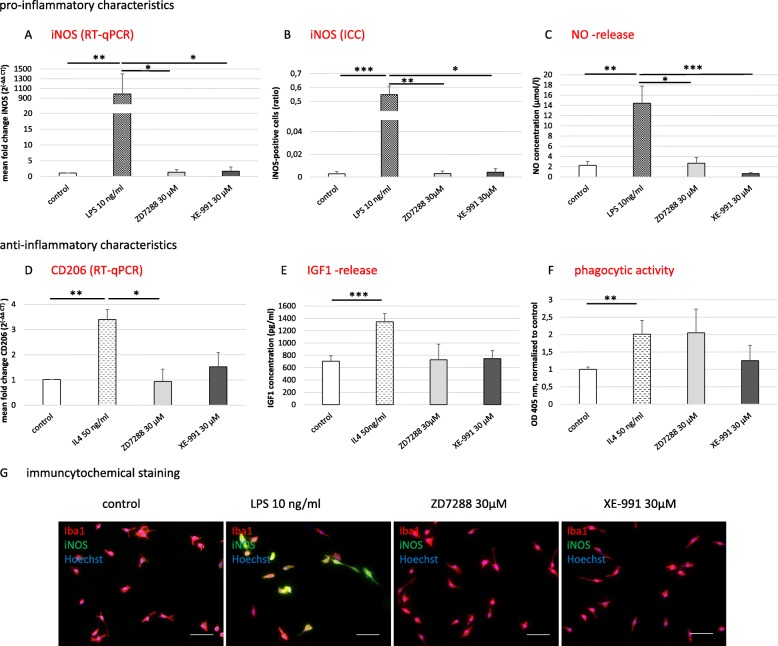
Activation characteristics of primary microglia. *p < 0.05; **p < 0.01; ***p < 0.001 compared to different experimental groups as marked by a horizontal bar. Characterization of the activated pro-inflammatory microglia phenotype by the expression of the inducible nitric oxide (NO) synthetase (iNOS) and release of NO after blockade of HCN and KCNQ/Kv7 channels with ZD7288 and XE-991 (30 μM each), respectively. Treatment with LPS (10 ng/ml) served as a positive control. **a**. iNOS expression was measured on RNA level by RT-qPCR (H(3) = 11.608, *p* < 0.009) and on **b** protein level by immunocytochemistry (H(3) = 17.665, *p* = 0.001). **c** Release of NO was measured by Griess assay (μmol/l; H(3) = 15.784, *p* = 0.001). Characterization of the anti-inflammatory phenotype of activated microglia by expression of CD206, release of insulin-like growth factor 1 (IGF1), and change of phagocytic capacity, after pharmacological block of HCN and KCNQ/Kv7 channels by 30 μM ZD7288 and XE-991, respectively. Treatment with IL4 (25 ng/ml) served as a positive control. **d** Regulation of CD206 expression on RNA level was measured by RT-qPCR (F (3, 20) = 13.981, *p* < 0.0001, *ω* = 0.62). **e** IGF1 release was measured by ELISA (pg/ml, H(3) = 15.805, *p* = 0.001). **f** Zymosan engulfment showed phagocytic activity of microglia (H(3) = 11.653, *p* < 0.009). Data were blank-corrected and normalized to control. **g** Representative immunocytochemical stainings for the microglia marker Iba1 (red), co-stained for iNOS (green), and Hoechst as a nuclear counterstain (blue); scale bars = 50 μm

Next, we assessed whether blockade of HCN and KCNQ/Kv7 channels resulted in microglia activation. To characterize the pro-inflammatory microglia phenotype, we conducted RT-qPCR as well as immunocytochemical staining for iNOS and investigated the release of NO by Griess assay. To characterize the anti-inflammatory phenotype, we assessed the expression of CD206 by RT-qPCR and the release of IGF1 by ELISA. The phagocytic potential was measured semi-quantitatively using zymosan particles. Recently we showed that LPS (10 ng/ml) boosts the pro-inflammatory characteristics and that IL4 (50 ng/ml) increases the expression of anti-inflammatory markers in primary microglia [17]. Therefore, LPS- and IL4-treated microglia served as the respective positive controls. The pharmacological blockade of HCN and KCNQ/Kv7 channels on primary microglia did not affect iNOS expression on RNA or protein levels as well as NO release compared to untreated controls (Fig. 4a–c, g). In parallel, exposure to ZD7288 or XE-991 did not affect the expression of CD206 or the release of IGF1 in comparison to untreated control microglia. Interestingly, there was a trend for increased phagocytic activity when HCN channels were blocked by ZD7288 at 30 μM (~ 2-fold compared to control, n.s., Fig. 4d–f).

### Microglia polarization requires functional HCN channels but not KCNQ/Kv7 channels

To further explore the impact of HCN and KCNQ/Kv7 channels on the dynamics of microglia polarization, we simultaneously exposed microglia to LPS or IL4 *plus* ZD7288 or XE-991, respectively. In order to impair the HCN channels with higher specificity, we transfected primary microglia with small interfering (si)RNA, which led to mRNA degradation of HCN2 transcripts (siHCN2-RNA), resulting in HCN2 gene silencing. Based on the predominant expression profile, as shown in Fig. 1a, we targeted HCN2. We confirmed the effect of siHCN2-RNA transfection by determination of significant reduction of HCN2-expression by RT-qPCR 24 h after transfection of resting, LPS, and IL4 treated microglia using two different primer pairs for HCN2 (supplemental fig. 3 A & B). In parallel, transfection with non-targeting siRNA (negative control siRNA) did not alter HCN2-expression and did not induce activation and polarization in primary microglia (supplemental fig. 3 A & B & supplemental fig. 4). Although the transfection procedure exerted moderate cell toxicity measured by live/dead assay and LDH release, we continued with experiments because cell counting still revealed at least > 65% living cells (supplemental fig. 3 C & D).

**Fig. 5:**
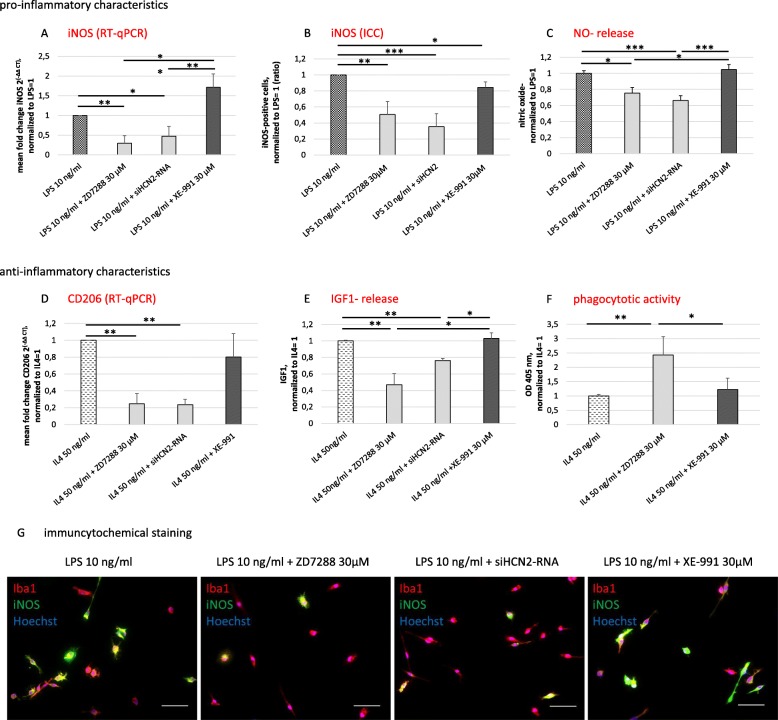
Impact of functional HCN and KCNQ/Kv7 channels on the activation capacity of primary microglia. *p < 0.05; **p < 0.01; ***p < 0.001. Stimulation of microglia with LPS (10 ng/ml) with simultaneous blockade of the HCN or the KCNQ/Kv7 channel and resulting expression of pro-inflammatory markers. The HCN channel was either blocked pharmacologically with ZD7288 (30 μM) or by transfection of silencer® small interfering (si)RNA targeting HCN2-mRNA (siHCN2-RNA). XE-991 (30 μM) was used to block KCNQ/Kv7. **a** Inducible nitric oxide (NO) synthase (iNOS) expression was measured on RNA level by RT-qPCR (H(3) = 17.142, *p* = 0.001) and on **b** protein level by immunocytochemistry (H(3) = 19.626, *p* < 0.0001). **c** NO release was measured by Griess assay (μmol/l; H(3) = 23.102, *p* < 0.0001). Data were normalized to LPS-only stimulation. Characterization of the anti-inflammatory microglia phenotype by the expression of CD206, release of insulin-like growth factor 1 (IGF1), and measurement of the phagocytic activity, after treatment with IL4 (50 ng/ml) and simultaneous blockade of the HCN and KCNQ/Kv7 channel as described above. **d** Regulation of CD206 expression was measured on RNA level by RT-qPCR (H(3) = 12.755, *p* = 0.005). **e** Release of IGF1 was measured by ELISA (pg/ml; H(3) = 16.611, p = 0.001). **f** Zymosan engulfment indicated phagocytotic activity of microglia (H(2) = 7.501, *p* = 0.024). Data were normalized to IL4-only stimulation. **g** Representative images of the immunocytochemical staining for the microglia marker Iba1 (red), co-stained for iNOS (green), and Hoechst as a nuclear counterstain (blue); scale bars = 50 μm

Simultaneous treatment with LPS plus pharmacological blockade by ZD7288 or transfection of siHCN2-RNA resulted in > 50% decrease of the expression of the pro-inflammatory marker iNOS on the RNA and protein level compared to LPS-only exposed microglia (*p* < 0.05 and *p* < 0.01, respectively, Fig. 5a, b, g). Correspondingly, the Griess assay revealed significantly less release of NO compared to LPS-only treated microglia (> 25%, *p* < 0.05, Fig. 5c). Similarly, simultaneous treatment with IL4 plus ZD7288 or transfection of siHCN2-RNA resulted in a substantial reduction in expression of the anti-inflammatory marker CD206 as compared to IL4-only exposed microglia (> 75%, *p* < 0.01, Fig. 5d). Likewise, the release of IGF1 was significantly diminished in comparison to IL4-only treated microglia (*p* < 0.01, Fig. 5e). Surprisingly, the simultaneous blockade of HCN channel by 30 μM ZD7288 resulted in a substantial enhancement of the phagocytic activity of IL4-stimulated microglia (*p* < 0.05, lower panel of Fig. 5f). On the contrary, blockade of KCNQ/Kv7 channel by XE-991 had neither an impact on microglia polarization potential upon LPS or IL4 treatment nor on the phagocytotic activity of primary microglia (Fig. 5).

Showing that ZD7288 mediated blockade of HCN channel activity and siRNA gene silencing of the HCN2 subunit counteracted the polarizing effects of LPS and IL4, our findings suggest that a functional HCN channel is required for the activation and polarization of primary microglia in vitro. Furthermore, the presented data suggest that HCN channels exert regulatory effects on the phagocytic activity of microglia.

## Discussion

This report is the first to show the expression of HCN and KCNQ/Kv7 channels on primary microglia in vitro. Our results demonstrate that in primary rat microglia, HCN channels have a significant regulatory impact on viability, proliferation, phagocytosis, and activation of microglia, while KCNQ/Kv7 channels solely affect migration (for summary of the findings compare Table 2). Our data confirm the presence of all HCN subunits as well as the Kv7 subunits Kv7.2, Kv7.3, and Kv7.5 on RNA and protein level in primary rat microglia. In 2014, Zhang and colleagues generated a transcriptome database of the different cell types in the mouse cerebral cortex [46], www.brainrnaseq.org/. Following this, our data reveal that HCN2 is expressed to a higher degree than other HCN subunits in primary microglia. Accordingly, our data confirm the expression of Kv7 channel subunits Kv7.2, Kv7.3, Kv7.5 in microglia. Notably, we did not observe any expression of the subunits Kv7.4 or Kv7.1 in primary microglia, consistent with previous reports describing their predominant expression in the mammalian heart and inner ear [43].

**Table 2:** Summary of the findings

A. Expression of HCN and KCNQ subunits in activated microglia compared to untreated microglia	
Pro-inflammatory microglia	Anti-inflammatory microglia
• HCN2 ↓HCN3 ↓ • KCNQ 3 ↑KCNQ 5 ↑	• HCN2 ↑HCN3 ↓ • KCNQ 3 ↓KCNQ 5 ↓
**B. Effects of HCN and Kv7/KCNQ channel blockade on microglia**	
**HCN-blockade**	**Kv7/KCNQ-blockade**
• survival ↑ • proliferation ↓ • activation ↓ • phagocytotic capacity ↑ • no effects on migration	• migration ↓ • no effects on survival, proliferation, phagocytotic capacity and activation

Reports about HCN channel expression in non-depolarizing cells—other than neurons and cardiomyocytes—are rare. Interestingly, Notomi et al. reported the presence and function of HCN channels in osteoclasts that—similar to microglia—perform phagocytosis, thereby contributing to cell homeostasis [37]. Therein, the authors describe that the I_h_ current in the osteoclast membrane is predominantly mediated by HCN4 subunits and regulates the promotion of osteoclast differentiation in the presence of zinc. Osteoclasts are of hematopoietic origin and differentiate from bone marrow macrophages [47]. On the contrary, in vivo lineage tracing studies established that adult parenchymal microglia derive from primitive myeloid progenitors arising before embryonic day 8, identifying microglia as an ontogenetically distinct population in the mononuclear phagocyte system [48, 49]. Nevertheless, microglia and macrophages share multiple properties, including marker expression patterns, as well as critical functions, such as the ability to preserve cell homeostasis by phagocytic activity and the capacity to polarize towards different activation phenotypes [50, 51].

Furthermore, our data reveal that the RNA expression of the HCN2 subunit is significantly downregulated in LPS-treated pro-inflammatory microglia, and upregulated in IL4-treated anti-inflammatory microglia. In contrast, pro-inflammatory microglia express significantly higher levels of Kv7.3 and Kv7.5 compared to the anti-inflammatory phenotype. These diametrical expression patterns of HCN and Kv7 channels might serve as markers to further characterize microglia polarization in the course of neuroinflammatory processes. Similarly, expression patterns of several inward and outward rectifying potassium channels were suggested to predict the activation phenotype of microglia [19, 20, 25–27, 52]. Among others, the voltage-gated potassium channel Kv1.3 was suggested as a marker for “classically” activated microglia in vitro upon stimulation with LPS or interferon-gamma [25], and in vivo data revealed a dramatically elevated expression of this channel in activated microglia of human brains with Alzheimer’s disease [52]. In contrast, expression of Kir2.1, an inward rectifying potassium channel, is exclusively enhanced after IL4 stimulation of microglia in vitro [25], resembling our observations on the HCN2 subunit.

Aside from recognizing markers that characterize microglia phenotypes, our research aims to identify molecular targets to regulate neuroinflammation in order to promote regeneration after CNS damage. Several ion channels on microglia were identified to play an essential role in microglia activation, and specific potassium channels were proposed as targets to modulate neuroinflammation [25, 27, 29, 53, 54]. Among those, Kv1.3 channels have received particular attention, since they are upregulated in LPS-treated microglia in vitro [25], in microglia acutely isolated from the infarct areas of mice subjected to experimental stroke [55], as well as in microglia located around the ß-amyloid plaque lesions in Alzheimer’s disease mouse model and in human brains affected by this neurodegenerative disorder [52, 53]. Blockade of the Kv1.3 channel in mouse models specifically inhibits distinct signal pathways participating in microglia activation [56] and enhances phagocytic activity of ß-amyloid plaques, resulting in a beneficial outcome with reduced neuroinflammation, a decrease in cerebral amyloid load, an enhancement of hippocampal neuronal plasticity, and an improvement of behavioral deficits [53]. In this context, the regulatory effects of HCN channels on viability, proliferation, phagocytosis, and activation potential demonstrated in the present study suggest HCN channels as possible therapeutic targets that merit further investigation.

Here we demonstrate that the blockade of HCN channels regulates not only the membrane potential but is also associated with a slow and sustained increase of the intracellular calcium concentration in subsets of primary microglia. Intracellular calcium concentration plays a significant role in microglia functions, including morphological changes, migration, proliferation, phagocytosis, as well as secretion of cytokines and reactive oxygen species (reviewed in [57, 58]). The majority of receptor agonists, as well as cation channels regulating membrane potentials, modulate intracellular calcium signaling, although via different mechanisms and with markedly different consequences (reviewed by [24]). For example, when LPS binds to the toll-like receptor 4 (TLR4) and mediates the activation of microglia towards the pro-inflammatory phenotype releasing cytokines and NO, the basal intracellular calcium concentration increases persistently [59]. Our experiments—demonstrating a comparable change in intracellular calcium concentrations—support the hypothesis that HCN channels are involved in the downstream intracellular signaling.

HCN channels have been extensively investigated in neurons and cardiomyocytes. They are activated by hyperpolarization and generate an excitatory inward current resulting in decreased input resistance or direct depolarization effect [60]. However, our data reveal that blockade of HCN currents with ZD7288 results in a reduced negativity of the membrane potential, increased conductance, and higher intracellular calcium levels. At least at first sight, these results appear to be a paradox because blockade of HCN currents is expected to result in hyperpolarization of the membrane, hence in a reduced intracellular cation level. However, several studies on neuronal cells describe a similar phenomenon: Enhancement of I_h_ currents by HCN channels results in hyperpolarization and a reduction of spike rates [61–64].

Conversely, downregulation of I_h_, e.g., through genetic deletion of HCN1 [65], pharmacological blockade using cesium [66, 67], or administration of ZD7288 [64, 66], increases the excitatory postsynaptic potential amplitude, temporal summation, and spike firing. Some data suggest that small changes in the resting membrane potential caused by HCN current may affect the activation of other ion channels. George et al. showed a direct relationship of the HCN currents with the regulation of concurrent KCNQ/Kv7 channels that can significantly counteract any HCN-mediated effects on the membrane potential of hippocampal pyramidal cells [68]. Others reported similar interactive regulation mechanisms of voltage-gated Ca2+ channels [69]. Both methods used in the present study, the voltage sensor probes, as well as the calcium imaging, screen the effects of ZD7288 in a considerable number of microglia, detecting even slight fluctuations of membrane potentials independent from the distribution of HCN channels in the plasma membrane. However, to unravel detailed electrophysiological changes after HCN channel blockade, further studies are warranted, e.g., single-cell patch-clamping.

In the present study, the blockade of the KCNQ/Kv7 channel with XE-991 did not exert a major impact on the function of microglia. Solely their migration was reduced. Remarkably, Romero et al. and Greene et al. revealed that XE-991 is less or even not sufficient to inhibit KCNQ/Kv7 channels in neurons, displaying reduced excitability and keeping a stable resting potential [70, 71]. Causatively, Greene and colleagues showed that XE-991 binds to a single activated subunit and, therefore, its efficacy is voltage-dependent [70], suggesting a reduced effect in non-excitable cells.

Assuming that microglia are key players in neuroinflammatory and neurodegenerative processes and that poor spontaneous recovery from cerebral injury is associated with the incorrect timing of microglia recruitment, excessive or insufficient numbers of microglia, and/or inappropriate microglia polarization [72], future therapeutic strategies might include the regulation of microglia function by targeting microglial HCN channels. In this context, the selective HCN blocker ivabradine—used in the treatment of angina pectoris and heart failure—was first approved for medical use by the European Medicines Agency in 2005 and later by the United States Food and Drug Administration in 2015 [73].

## Conclusion

Taken together, our data suggest that at least HCN channels could be an essential player and a potential target to modulate neuroinflammatory processes. Further studies are required to (i) decipher electrophysiological effects as well as intracellular downstream effects of HCN channels in primary microglia and (ii) reveal their role on microglia in vivo in health and disease. The fact that the HCN channel blocker ivabradine was recently approved for the treatment of cardiac disorders bestows an immediate clinical relevance of the presented data.

## Supplementary information

**Additional file 1: Supplemental Figure 1.** Microglia express subunits of HCN and KCNQ/Kv7 channels. Representative images of primary microglia show staining of Iba1 (red) and HCN-subunits 1, 2, 3, or 4, or the Kv7.2 and Kv7.3-subunit (green). Hoechst stains all cell nuclei blue. Negative control was conducted without primary antibody. Images were taken with fluorescence microscope, scale bar 10 μm.

**Additional file 2: Supplemental Figure 2.** Representative images of traces of intracellular calcium responses of individual microglia. For Ca^**2**+^ imaging, cells were loaded with Fluo-4. Ca^2+^ responses of individual cells were examined in the presence of 30 μM ZD7288 (after 201 seconds of baseline measurement) and subsequent ATP-application (200 μM, 300 seconds after ZD7288 application). A. About 41% of all microglia followed (38/92) did not show any changes of intracellular calcium concentration ([Ca^2+^]_i_) upon ZD7288 application, although ATP led to high [Ca^2+^]_i_. B. The majority of all followed microglia (>50%) showed a constant slow increase of [Ca^2+^]i. Single cells revealed different calcium responses than described in A and B upon treatment with ZD7288: C. Ca^2+^ peak with slow reduction of [Ca^2+^]_i_,. D. Ca^2+^ oscillations or Ca2+ peaks on top of an elevated Ca2+ level or. E. Ca^2+^ peak with rapid reduction of [Ca^2+^]_i._

**Additional file 3: Supplemental Figure 3.** Effect of small interfering RNA transfection on primary microglia. *: p< 0.05; ** p< 0.01; *** p< 0.001. A. Capacity of transfected *silencer*® small interfering (si)RNA to induce degradation of the mRNA of HCN2 (siHCN2-RNA) in primary microglia. *Silencer*® select negative control siRNA, a non-targeting negative control siRNA served as control. Microglia were untreated or activated with LPS (10 ng/ml) or IL4 (50 ng/ml). Expression levels of the HCN2-RNA were revealed by RT-qPCR. Two different primer pairs for HCN2 were used (upper and lower panel). B. Toxicity of the transfection procedure was measured by live/dead-assay. Microglia were transfected with siHCN2-RNA or negative control siRNA and simultaneously treated with LPS (10 ng/ml) or IL4 (50 ng/ml). Ratio of viable versus dead (propidium iodide-positive) microglia was quantified 24 hours after treatment (n= 3, H(8)= 26.714, p= 0.001). C. Release of lactate dehydrogenase (LDH) was measured photometrically (LDH-assay) as a surrogate for cell death after treatment of microglia under the same conditions as outlined in B (n= 3, H(8)= 25.313, p= 0.001).

**Additional file 4: Supplemental figure 4.** Characterization of the pro- and anti-inflammatory microglia phenotype by expression of inducible nitric oxide (NO)-synthetase (iNOS), CD206, and release of NO and insulin-like growth factor 1 (IGF1) after transfection of *Silencer*® select negative control siRNA (all data were not statistically significant).

## Data Availability

All data generated or analyzed during this study are included in this published article and its supplementary information files.

## Abbreviations

CNS: Central nervous system
FCS: Fetal calf serum
FRET: Fluorescence resonance energy transfer
HCN: Hyperpolarization-activated cyclic nucleotide-gated
iNOS: Inducible nitric oxide synthase
IL: Interleukin
LDH: Lactate dehydrogenase
LPS: Lipopolysaccharides
NSC: Neural stem cell
NO: Nitric oxide
OD: Optical density
PBS: Phosphate-buffered saline
RT-qPCR: Real-time quantitative PCR
si: Small interfering
DMEM: Dulbecco’s modified eagle medium
PFA: Paraformaldehyde
VSP: Voltage-sensor probe
