# Supplementary figures and images for: The impact of hyperpolarization-activated cyclic nucleotide-gated (HCN) and voltage-gated potassium KCNQ/Kv7 channels on primary microglia function
Source: J Neuroinflammation. 2020 Apr 6;17:100. doi: 10.1186/s12974-020-01779-4 (PMC7132998; doi:10.1186/s12974-020-01779-4)

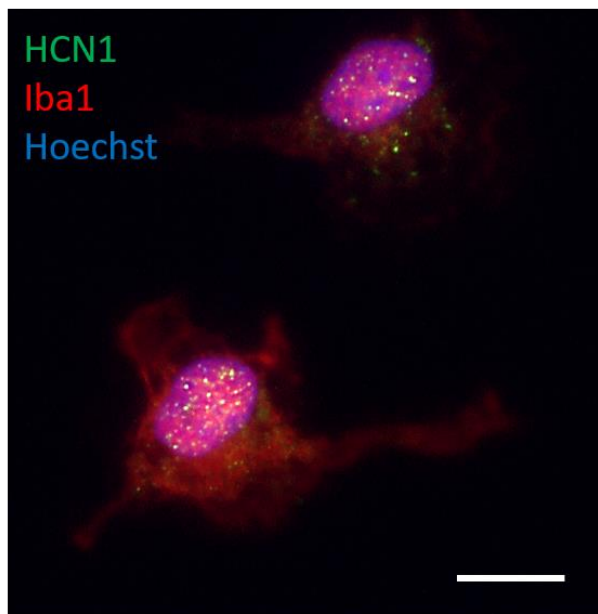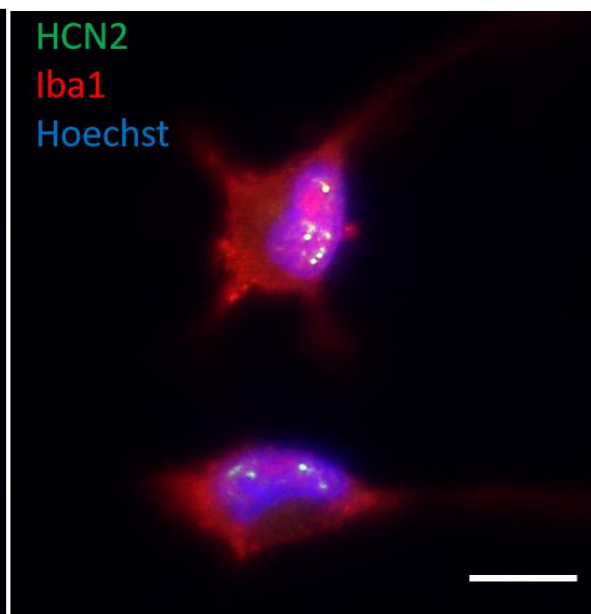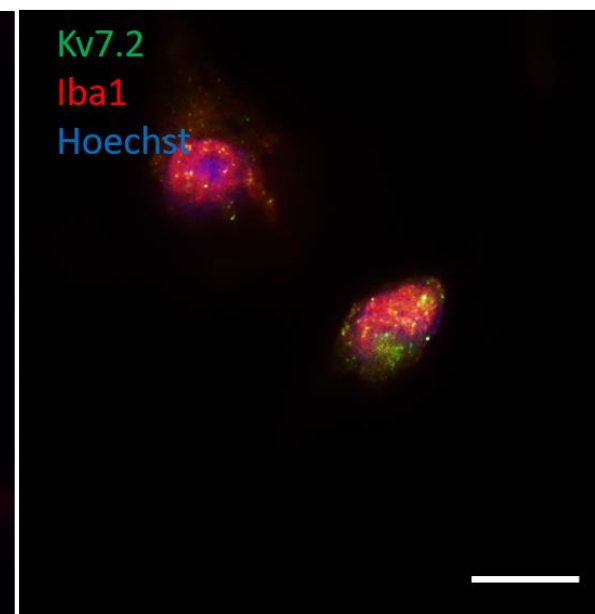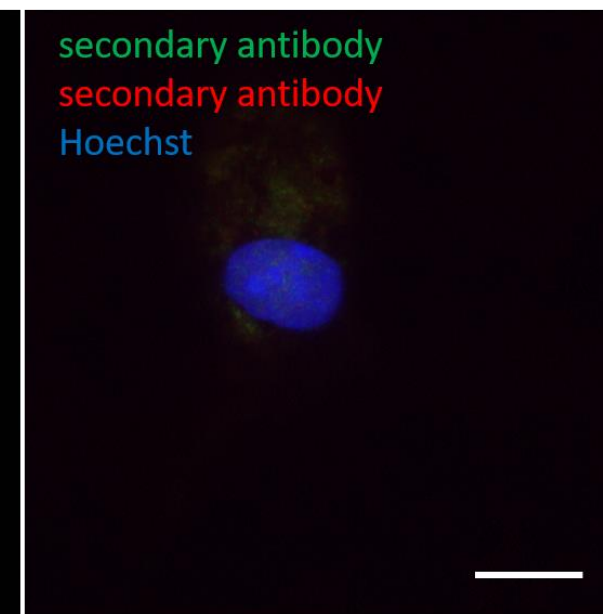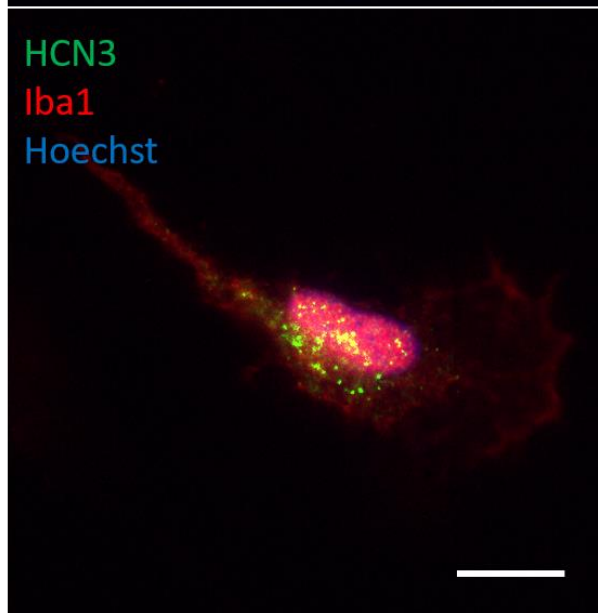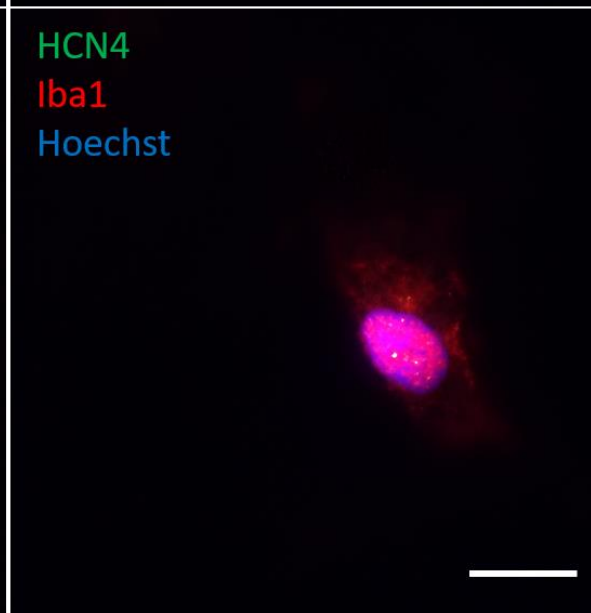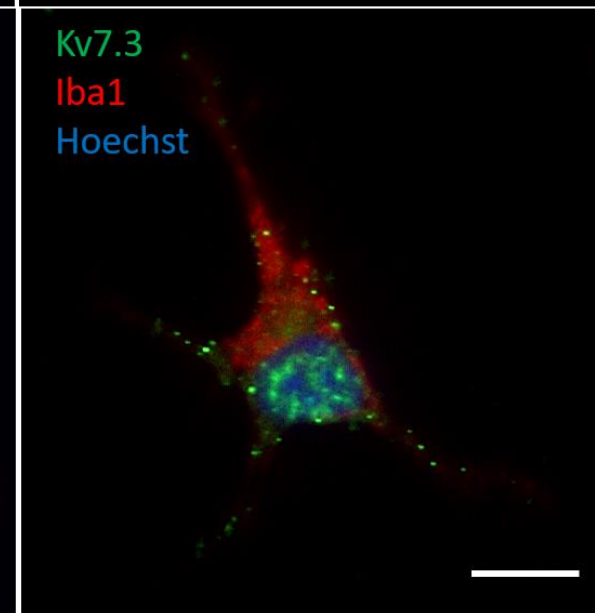

Supplement: Supplementary file 1 — Additional file 1: Supplemental Figure 1. Microglia express subunits of HCN and KCNQ/Kv7 channels. Representative images of primary microglia show staining of Iba1 (red) and HCN-subunits 1, 2, 3, or 4, or the Kv7.2 and Kv7.3-subunit (green). Hoechst stains all cell nuclei blue. Negative control was conducted without primary antibody. Images were taken with fluorescence microscope, scale bar 10 μm. [file 12974_2020_1779_MOESM1_ESM.pdf]

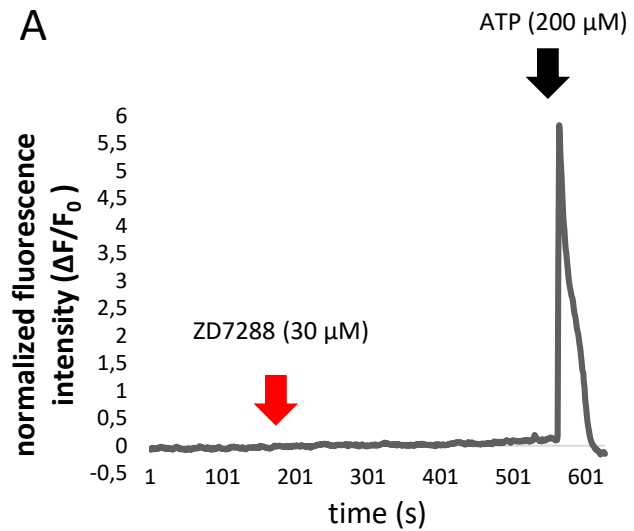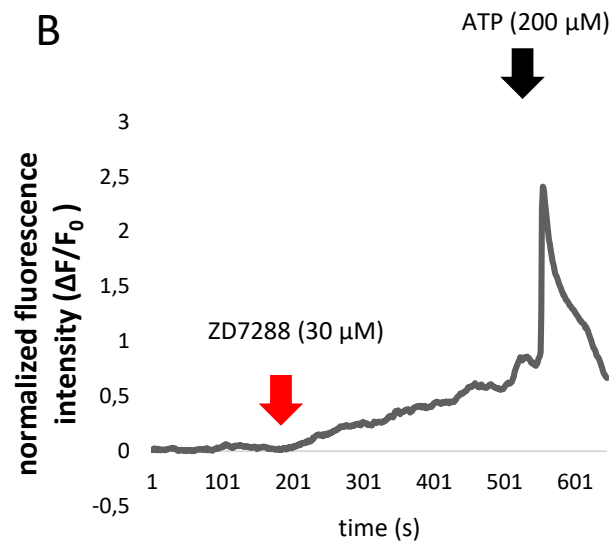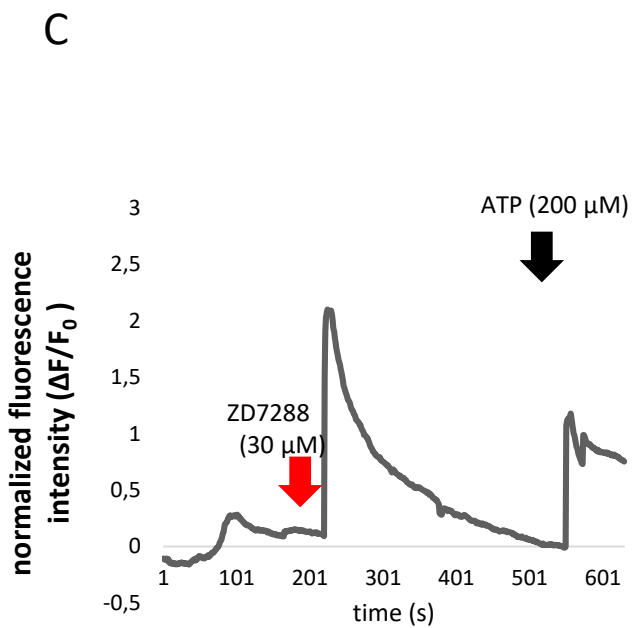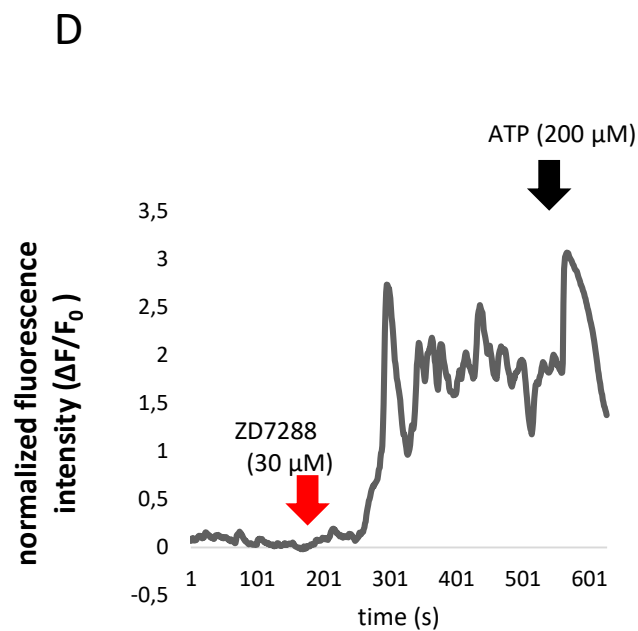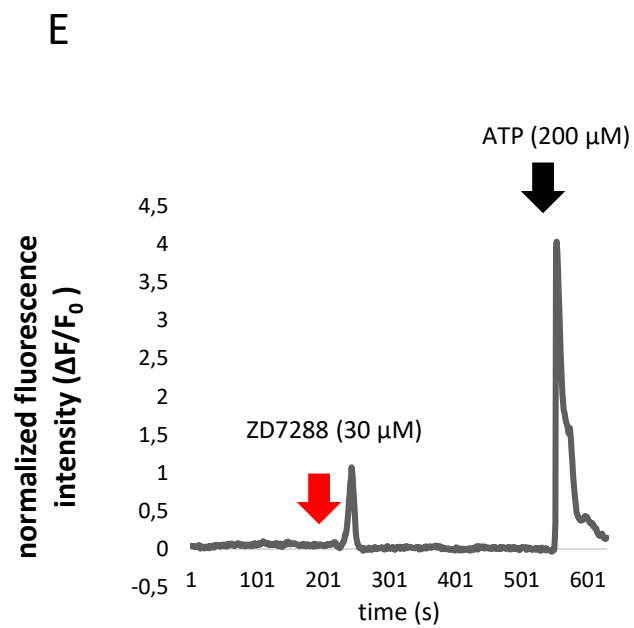

Supplement: Supplementary file 2 — Additional file 2: Supplemental Figure 2. Representative images of traces of intracellular calcium responses of individual microglia. For Ca2+ imaging, cells were loaded with Fluo-4. Ca2+ responses of individual cells were examined in the presence of 30 μM ZD7288 (after 201 seconds of baseline measurement) and subsequent ATP-application (200 μM, 300 seconds after ZD7288 application). A. About 41% of all microglia followed (38/92) did not show any changes of intracellular calcium concentration ([Ca2+]i) upon ZD7288 application, although ATP led to high [Ca2+]i. B. The majority of all followed microglia (>50%) showed a constant slow increase of [Ca2+]i. Single cells revealed different calcium responses than described in A and B upon treatment with ZD7288: C. Ca2+ peak with slow reduction of [Ca2+]i,. D. Ca2+ oscillations or Ca2+ peaks on top of an elevated Ca2+ level or. E. Ca2+ peak with rapid reduction of [Ca2+]i. [file 12974_2020_1779_MOESM2_ESM.pdf]

### A HCN2 –Primer No° 1

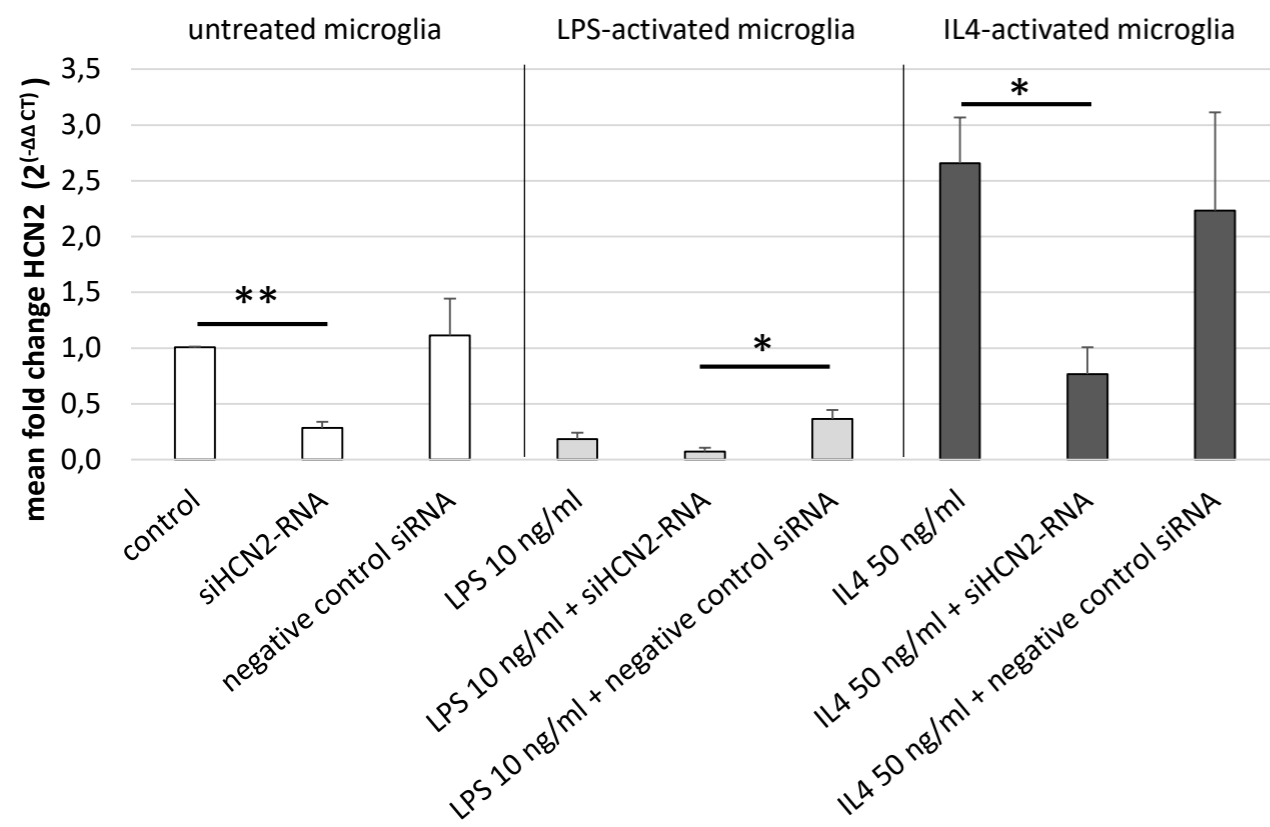

### B HCN2 –Primer No° 2

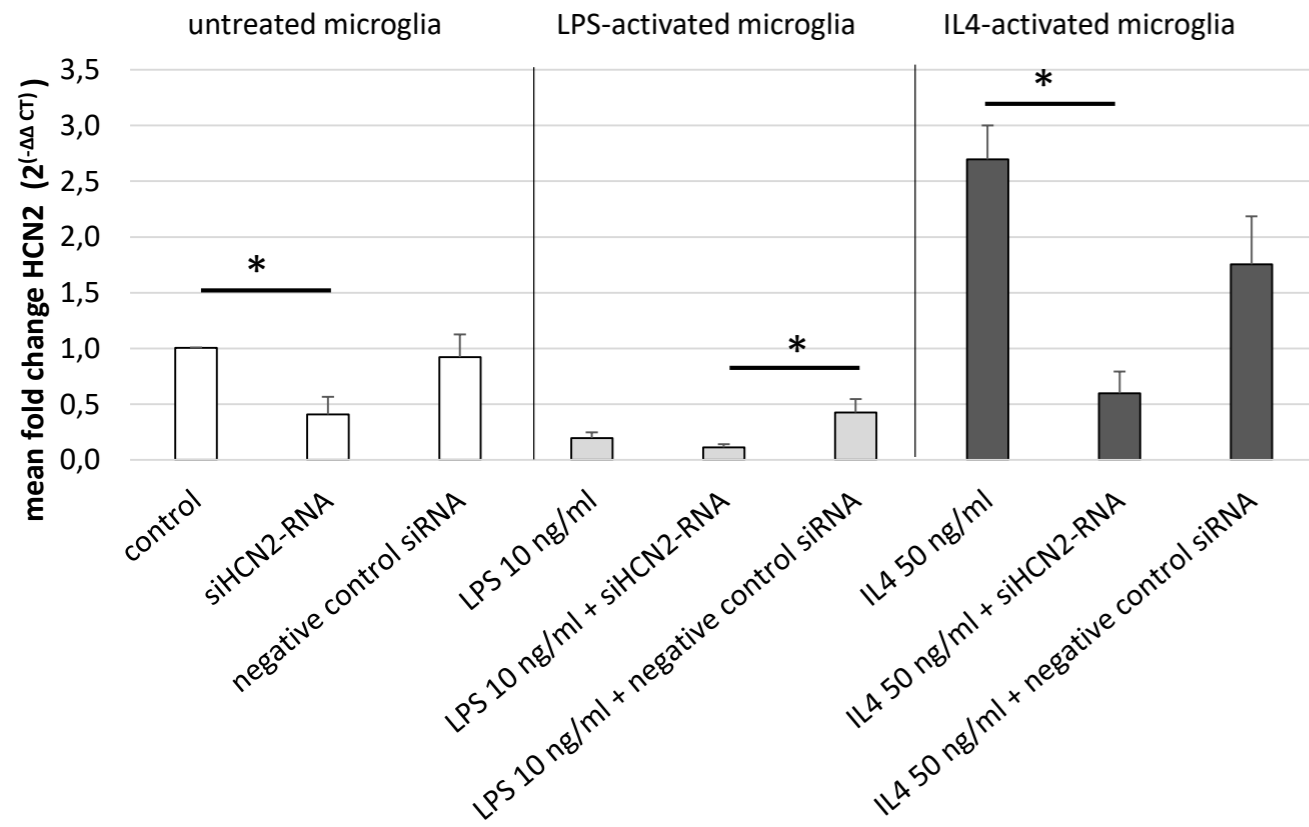

### C live-dead-assay

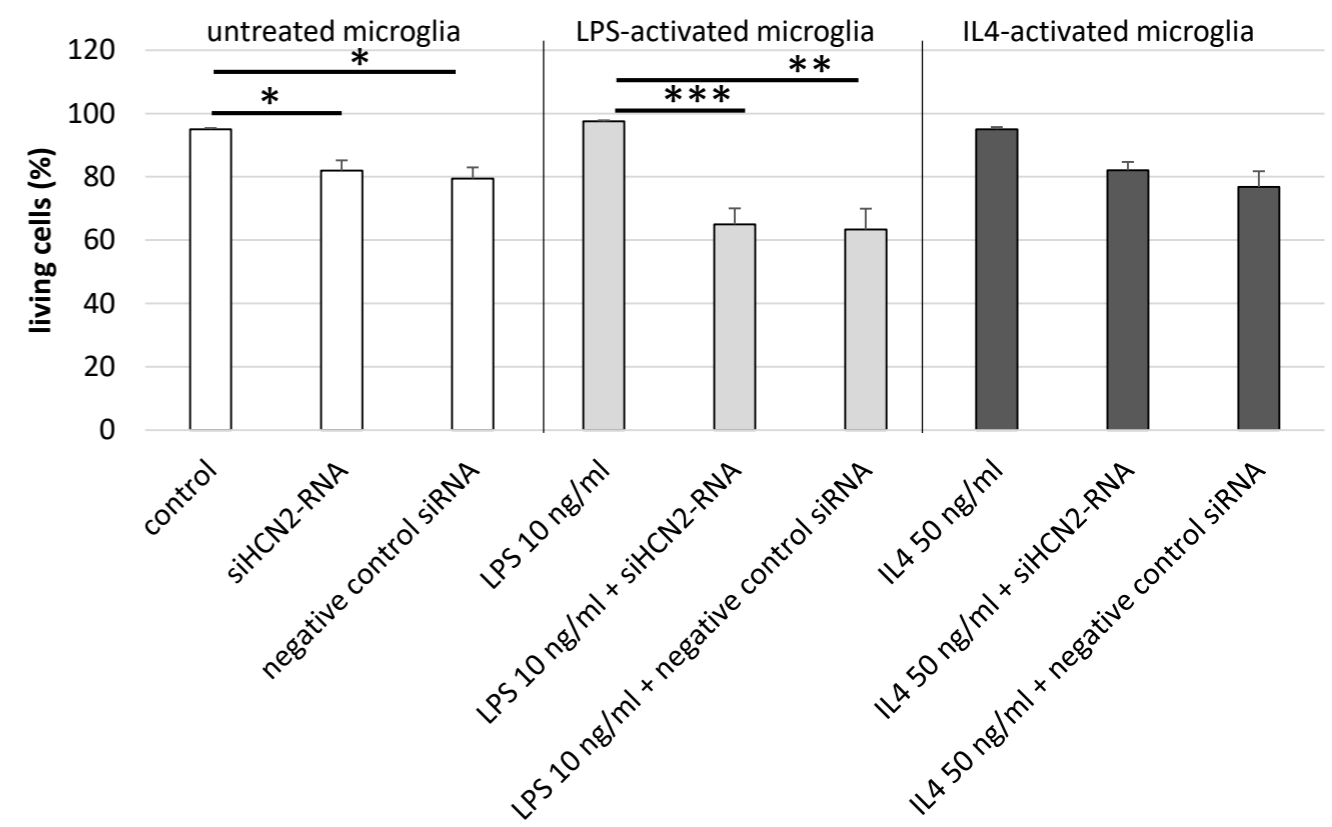

### D LDH-release

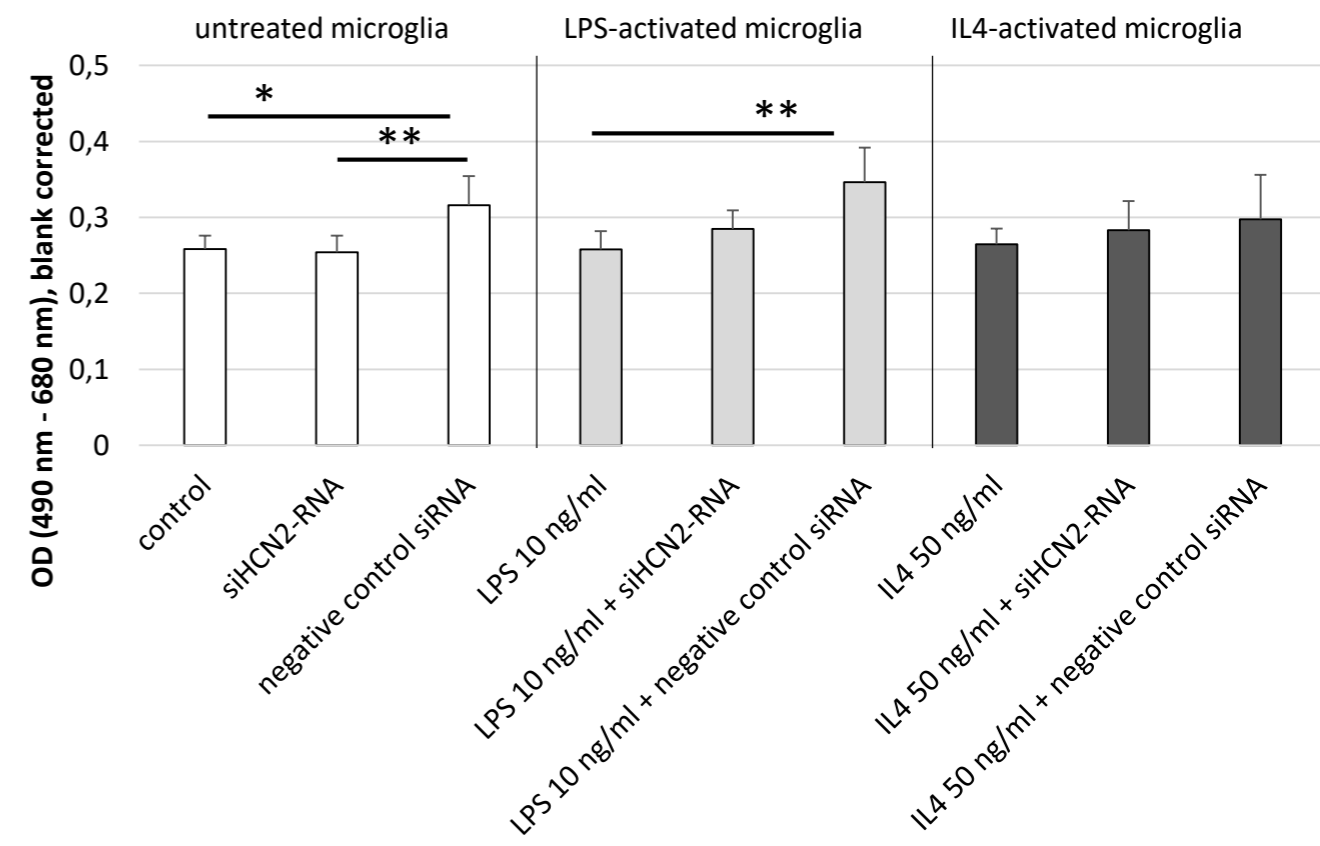

Supplement: Supplementary file 3 — Additional file 3: Supplemental Figure 3. Effect of small interfering RNA transfection on primary microglia. *: p< 0.05; ** p< 0.01; *** p< 0.001. A. Capacity of transfected silencer® small interfering (si)RNA to induce degradation of the mRNA of HCN2 (siHCN2-RNA) in primary microglia. Silencer® select negative control siRNA, a non-targeting negative control siRNA served as control. Microglia were untreated or activated with LPS (10 ng/ml) or IL4 (50 ng/ml). Expression levels of the HCN2-RNA were revealed by RT-qPCR. Two different primer pairs for HCN2 were used (upper and lower panel). B. Toxicity of the transfection procedure was measured by live/dead-assay. Microglia were transfected with siHCN2-RNA or negative control siRNA and simultaneously treated with LPS (10 ng/ml) or IL4 (50 ng/ml). Ratio of viable versus dead (propidium iodide-positive) microglia was quantified 24 hours after treatment (n= 3, H(8)= 26.714, p= 0.001). C. Release of lactate dehydrogenase (LDH) was measured photometrically (LDH-assay) as a surrogate for cell death after treatment of microglia under the same conditions as outlined in B (n= 3, H(8)= 25.313, p= 0.001). [file 12974_2020_1779_MOESM3_ESM.pdf]

### iNOS (RT-qPCR)

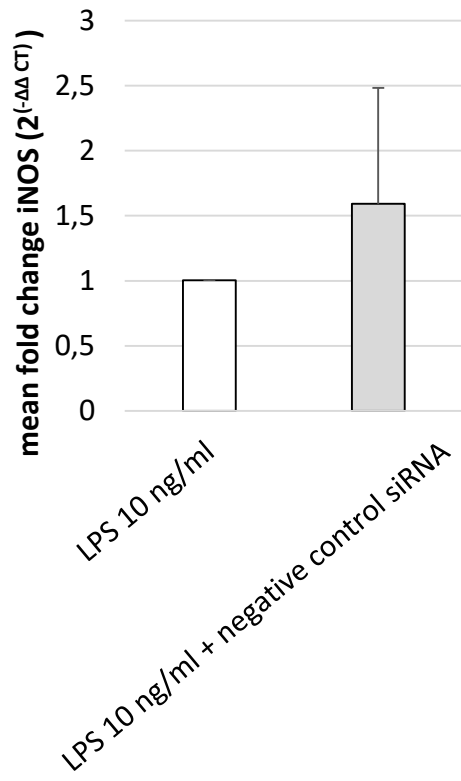

### NO

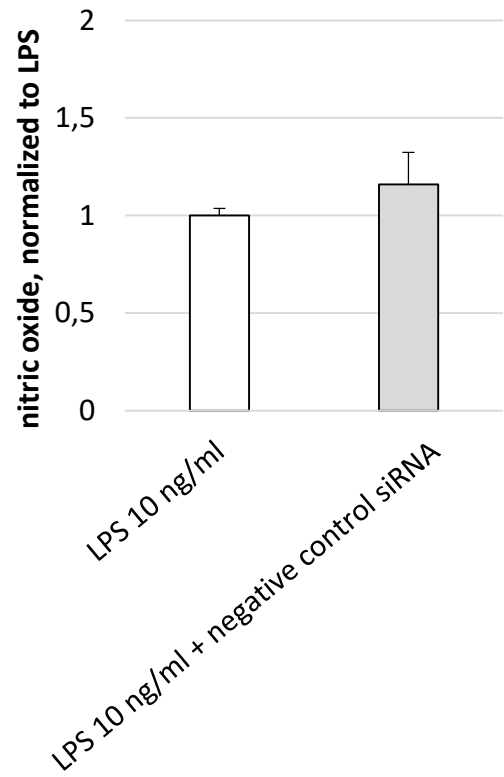

### CD206 (RT-qPCR)

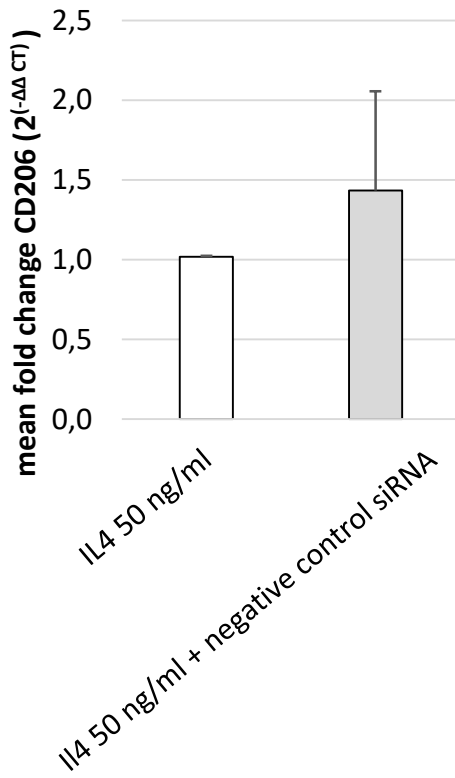

### IGF1

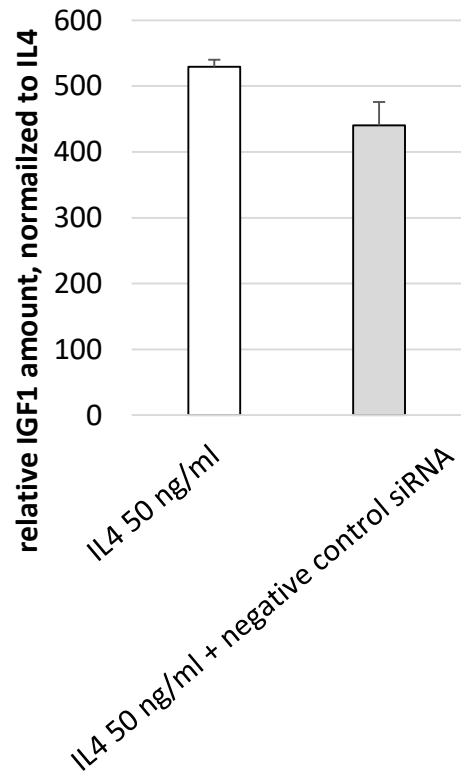

Supplement: Supplementary file 4 — Additional file 4: Supplemental figure 4. Characterization of the pro- and anti-inflammatory microglia phenotype by expression of inducible nitric oxide (NO)-synthetase (iNOS), CD206, and release of NO and insulin-like growth factor 1 (IGF1) after transfection of Silencer® select negative control siRNA (all data were not statistically significant). [file 12974_2020_1779_MOESM4_ESM.pdf]
